# The potential of residual clinical Group B *Streptococcus* swabs for assessing the vaginorectal microbiome in late pregnancy

**DOI:** 10.1038/s41598-024-70431-5

**Published:** 2024-08-20

**Authors:** Laura K. Boelsen, Melanie J. Williams, Yeukai TM. Mangwiro, Herah Hansji, Anna Czajko, Vanessa Marcelino, Samuel Forster, Joanne M. Said, Catherine Satzke, Richard Saffery

**Affiliations:** 1 Murdoch Children’s Research Institute, RoyalChildren’sHospital, Parkville, VIC Australia; 2 Centre for Innate Immunity and Infectious Diseases, Hudson Institute of Medical Research, Clayton, VIC Australia; 3 Department of Molecular and Translational Sciences, Monash University, Clayton, VIC Australia; 4 Department of Obstetrics and Gynaecology, Melbourne Medical School, The University of Melbourne, Melbourne, Australia; 5 Department of Maternal-Fetal Medicine, Joan Kirner Women’s & Children’s at Sunshine Hospital, Western Health, Melbourne, Australia; 6 Department of Paediatrics, The University of Melbourne, Parkville, VIC Australia; 7 Department of Microbiology and Immunology, The University of Melbourne at the Peter Doherty Institute for Infection and Immunity, Parkville, VIC Australia

**Keywords:** Medical research, Microbiome

## Abstract

The maternal pregnancy microbiome (including genitourinary and gut) has been linked to important pregnancy/birth and later childhood health outcomes. However, such sampling as part of large population cohort studies is logistically and financially challenging. Many countries routinely collect vaginal or vaginal-rectal swabs in late pregnancy for Group B Streptococcus (GBS) screening, but their utility for population-based research is still unclear. As part of planning for the Generation Victoria population-based cohort study beginning in pregnancy, we assessed the utility and reliability of residual clinical GBS vaginal/vaginal-rectal swabs for generating late pregnancy microbiome data. We carried out a two-phased pilot study. Phase one assessed the level of microbial diversity apparent in ‘residual’ clinical vaginal/vaginal-rectal swabs post clinical testing and storage for 7–10 days at 4 °C (routine clinical practice). Phase two directly assessed the impact of storage time and temperature on the microbial composition of vaginal/vaginal-rectal swabs collected specifically for research purposes. The microbiota composition in the ‘residual’ clinical swabs aligned with published studies. The ‘research’ swabs, stored at 4 °C for up to ten days, showed minimal changes in microbiota profile, compared to swabs examined on the day of collection. In contrast, significant variation in diversity was seen in swabs stored at room temperature for up to 48 h. Residual clinical material from swabs collected primarily for GBS screening in late pregnancy represent a reliable and abundant source of material for assessing the late pregnancy maternal microbiome for research purposes. This represents a low-burden opportunity for population-representative pregnancy studies to assess the potential of late pregnancy microbiome for prediction and understanding maternal and child health outcomes.

## Introduction

Group B Streptococcus (GBS, *Streptococcus agalactiae*) is an opportunistic Gram-positive pathogen commonly found in the gastrointestinal and genitourinary tracts of healthy individuals. Due to its status as a primary cause of neonatal infections worldwide^1^, the Centers for Disease Control and Prevention (CDC) recommended universal screening of all pregnant women for GBS in 1996^2,3^. In Australia, both routine universal antenatal GBS screening (by the collection of a vaginal-rectal swab at 35–37 weeks gestation) and antibiotic treatment during labour (based on a risk-factor assessment in the absence of universal screening) are currently offered in clinical practice^4^.

Studies of vaginal microbiota have found at least six different compositional profiles or ‘community state types’ (CSTs), four of which were dominated by a different *Lactobacillus* species^5,6^. In pregnancy there is a shift in microbiota to a more stable and less diverse state often dominated by one of these *Lactobacillus* species^7^. The ‘typical’ vaginal microbiome has been found to differ by race and ethnic background, including the predominant *Lactobacillus* species observed in pregnancy^5,6^.

In addition to the clear link between maternal GBS carriage and adverse offspring outcomes, the broader maternal microbiome, specifically the vaginal microbiome, has contributed to an understanding of how microbial markers for preterm birth vary across populations^8^. However, additional, larger studies are required to more comprehensively test the link between the late pregnancy vaginal microbiome and pregnancy outcome. Large studies of this nature are rare, primarily due to the practicalities and cost of large-scale research collection of samples for microbiome analysis. As such, the clinical relevance of the maternal microbiome, especially during late pregnancy, is still unclear.

Thus, maternal and child health could benefit from a ready and inexpensive mechanism to enable large, truly population-representative studies of the maternal pregnancy microbiome. Vaginal or vaginal-rectal swabs are routinely collected in late pregnancy for GBS testing, after which the swabs are discarded. As part of our feasibility studies for a large population-wide life course study (Generation Victoria; GenV), we aimed to test the utility of residual clinical GBS swab transport media for the generation of late pregnancy microbiome profiles. We also assessed the impact of storage time and temperature on the stability of these profiles.

## Materials and methods

### Samples and study design

Clinical guidance for sample collection recommends either a clinician-collected or patient-self- collected combined vaginal-rectal swab at 35–37 weeks gestation, using one single dry swab inserted into the vaginal introitus, then inserted into the anus. The swab should then be placed into standard bacterial transport medium^9^. Current recommendations from the CDC state to place the swabs into a non-nutritive transport medium in which GBS isolates remain viable for up to four days at room temperature^10^. Currently in Australia, either a liquid Amies, or a gel medium is used for transport of swabs to pathology labs. The liquid Amies transport medium is most widely used and is specifically formulated to maintain viability of a wide range of microbial organisms. This was therefore the focus of the current study.

**Figure 1:**
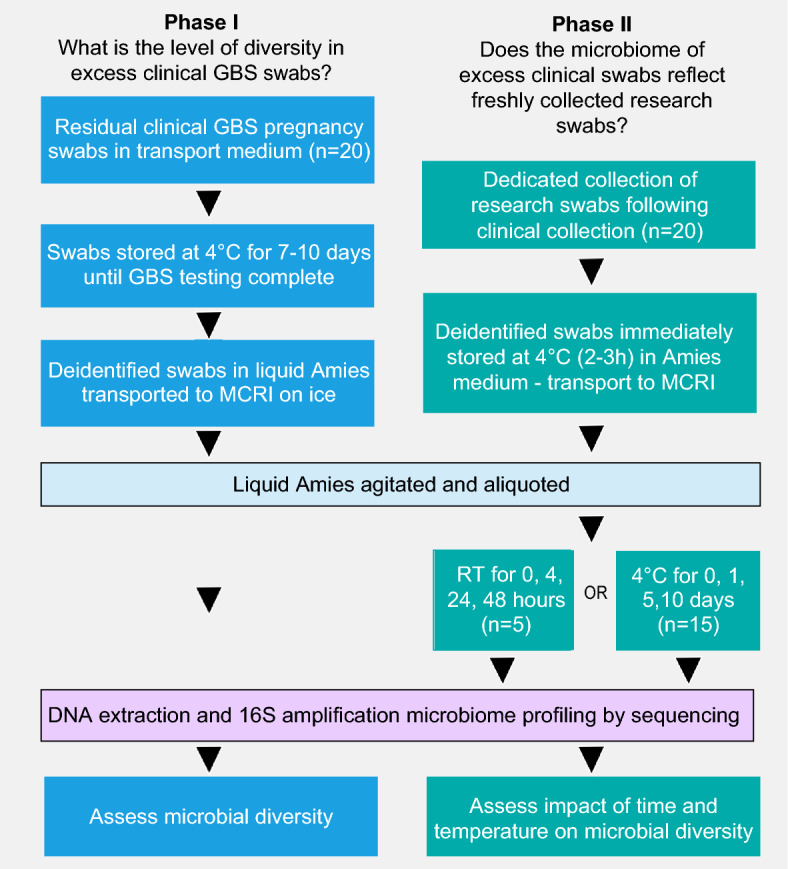
The journey and experimental process of GBS swabs collected for Phases I and II of the pilot study. Phase I illustrates the journey of vaginal and vaginal-rectal swabs following clinical testing for GBS and mandatory storage for seven days. Phase II illustrates the journey of dedicated vaginal and vaginal-rectal swabs collected for the purpose of this Piot study. Phase II samples were stored at either room temperature (RT) or 4 °C.

This pilot study had two phases (Fig. 1). The first involved assessing the level of diversity of microbial species present on vaginal and vaginal-rectal swabs following clinical testing for GBS and mandatory storage for seven days. Once collected and tested as part of routine antenatal care, these swabs are typically stored for seven days at 4 °C prior to being discarded. These ‘residual’ clinical swabs (n = 20) were obtained from Austin Pathology, Victoria, Australia seven to ten days after collection and stored at 4 °C until received at Murdoch Children’s Research Institute (MCRI). Following completion of clinical testing, swabs were transported to MCRI on ice, then processed the same day. The swabs and storage media were vortexed for 30 s and the swab was pressed against the side of the tube to remove residual liquid before disposal. The storage media was vortexed again for another 30 s. An average of 750 μL was collected and each sample was split into three aliquots of 250 μL adequate for a maximum of three DNA extractions if required. Aliquots were centrifuged for 5 min at 14,000 × *g* at room temperature, with the supernatant discarded prior to storage of the pellet at− 30 °C. A blank swab was processed alongside clinical swabs to act as a negative control for each batch of clinical swabs processed.

In the second phase, dedicated vaginal/vaginal-rectal research swabs were self-collected alongside routine clinical swabs for GBS screening from 20 pregnant women attending the antenatal clinics at Sunshine Hospital, Victoria, Australia. Most of the samples were vaginal only (13/20; 65%) and the remainder were combined vaginal-rectal samples (7/20; 35%). One sample was clinician collected (1/20; 5%) rather than self-collected (19/20; 95%). The instructions given to participants were the same as for clinical sample collection, to insert the flocked swab (Copan ESwab^™^, catalogue no. 480 °C) 2 cm into the vagina and then 1 cm into the anus before placing the swab into 1 mL of liquid Amies media. At the time of collection, the following were recorded: estimated delivery date, current gestation, date and time of swab collection, specimen collector (clinician or participant, and the site/s (i.e., vaginal, or vaginal-rectal). Immediately after collection, Amies media containing swabs were stored at 4 °C (for approximately 2–3 h) before transport on ice to MCRI for processing. Amies samples with swabs were tested under two sets of conditions: n = 15 samples were stored at 4 °C with 230 μL aliquots taken on days 0, 1, 5, and 10; n = 5 swabs were kept at room temperature with 230 μL aliquots taken at timepoints 0 h (receipt at MCRI), 4 h, 24 h, and 48 h. In all instances, the swab was removed from Amies media when the first aliquot was taken, with a 30 s vortex step before and after swab removal. The media was then returned to allocated storage conditions, with additional 30 s vortex prior to each subsequent aliquot. A blank control swab was also processed under each of the conditions. All aliquots were then processed as described for phase one.

### DNA extractions

Bacterial DNA was extracted using the Qiagen QIAamp DNA Mini kit and a modified version of the manufacturer’s Appendix D (Isolation of genomic DNA from Gram-positive bacteria) instructions. Briefly, pellets were resuspended in 180 μL of enzymatic lysis buffer (containing final concentrations of 20 mM Tris–HCl, 2 mM Na-EDTA, 1% v/v Triton X-100, 2 mg/mL RNase A, 0·075 mg/ml mutanolysin (M9901, Sigma-Aldrich) and 20 mg/mL lysozyme (L6876, Sigma-Aldrich)) and incubated at 37 °C for 60 min. Following incubation, 10 μL of 20% v/v SDS was added followed by 20 μL proteinase K and 200 μL of buffer AL with incubation at 56 °C for 60 min. An additional 200 μL of buffer AL was then added before a short incubation at 70 °C for 10 min. DNA was eluted in buffer EB after a 3 min incubation at.

room temperature and reapplication of eluate to spin-column. A blank reagent-only extraction control was included in each extraction batch.

### 16S rRNA PCR and sequencing

Microbial DNA was amplified by PCR targeting the V1-V2 hypervariable regions of the 16S rRNA gene in line with earlier maternal microbiota studies^6,8,11^. Amplifications were conducted in duplicate 25 μL reactions containing 5 μL of template DNA, 1X Q5® high-fidelity 2X master mix (M0492, New England BioLabs), and 500 nM each of forward and reverse primers (Sigma-Aldrich) containing Illumina adaptor sequences for downstream sequencing (27F 5’-TCGTCGGCAGCGTCAGATGTGTATAAGAGACAGAGMGTTYGATYMTGGCTCAG-3’ and 338R 5’-GTCTCGTGGGCTCGGAGATGTGTATAAGAGACAGGCTGCCTCCCGTAGGAGT-3’, respectively with adaptor sequences underlined). Cycling conditions^12^ were denaturation at 98 °C for 30 s, 20 cycles of 98 °C for 10 s, 66 °C for 20 s (reducing by 0·8 °C per cycle) and 72 °C for 20 s, before a final extension at 72 °C for 2 min. Cycling conditions were selected to balance adequate amplification of microbiota, and minimising the amplification bias and amplification of potential kit contaminants. All amplification runs included a no template control, a positive control (containing a mix of DNA previously extracted from pure cultures of *Streptococcus pneumoniae*, *Streptococcus agalactiae* (GBS), *Staphylococcus aureus*, *Escherichia coli*, *Klebsiella pneumoniae* and *Lactiplantibacillus plantarum*) and the controls from earlier steps (blank swab controls and extraction controls).

Following amplification by PCR, samples and controls underwent library preparation and quality control following the Illumina ‘16S Metagenomic Sequencing Library Preparation’ workflow (Document # 15044223 Rev. B) and the Nextera library preparation kit. Samples and controls were then sequenced by the MCRI Genomics Centre using paired end 2 × 250 bp (V2 reagent kit, Illumina) on MiSeq sequencing platform (Illumina).

### Sequence processing and data analysis

All sequences were processed using R (https://www.R-project.org/; version 4.1.1) and the DADA2 package (version 1.20.0)^13^ based on the DADA2 Pipeline Tutorial (1.16) (https://benjjneb.github.io/dada2/tutorial.html). Sequence processing parameters which were modified from the default in the tutorial included truncating forward and reverse reads at 240 bp, removal of primer sequences, and a maximum expected error of two allowed for forward reads and four for reverse reads. After denoising and merging the forward and reverse reads, sequences shorter than 300 bp or longer than 330 bp were removed. Taxonomy was assigned to amplicon sequence variants (ASVs) initially using the DADA2-formatted Silva reference database (version 138.1)^14^ with taxonomy assignments checked against the DADA2-formatted RDP trainset 18 reference database^15^, and the ‘16S ribosomal RNA’ BLAST database with BLAST + ^16^. Discrepancies in nomenclature were resolved using the NCBI taxonomy browser^17^.

After sequence processing and taxonomic assignment, data were analysed using R packages except where indicated (see supplementary material for full code). Alpha-diversity was determined using Phyloseq (version 1.36.0)^18^ with the ‘estimate_richness’ function. R packages Phangorn (version 2.7.1)^19^ and msa (version 1.24.0)^20^ were used to generate a phylogenetic tree using ‘dist.ml’ and ‘NJ’ functions with model fitting using ‘pml’ and ‘optim.pml’. Distance matrices for beta-diversity analyses were generated using the ‘distance’ function (using ‘bray’, ‘jaccard’,’unfrac’, and ‘wunifrac’ methods) of Phyloseq along with ‘ordinate’ to generate ordination plots (‘NMDS’ and ‘PCoA’ methods). Differences in bacterial composition were assessed using the distance matrices and PERMANOVA with the Vegan package (version 2.5–7)^21^ and the ‘adonis’ function. The differential relative abundance between time points analyses were done using DESeq2 (version 1.32.0)^22^. Graphs were generated using a combination of Phyloseq and ggplot2 (version 3.3.5)^23^. Random forest analyses were done using the randomForest (version 4.7.1)^24^ and rfUtilities (version 2.1–5)^25^. Data analysis was also performed using GraphPad Prism (https://www.graphpad.com/; version 9.3.1 for Windows, GraphPad Software, San Diego, California USA).

### Statistics

Mean and standard deviation are supplied where data were normally distributed (after passing “omnibus K2” D’Agostino & Pearson test), otherwise median and interquartile range (IQR) are provided. For differential relative abundance using DESeq2, an adjusted p-value (corrected for multiple testing using the Benjamini and Hochberg method) of < 0·05 was considered statistically significant.

### Sequence data availability

The dataset generated and analysed in the current study is available in the NCBI Sequence Read Archive, accession number PRJNA929552 (http://www.ncbi.nlm.nih.gov/bioproject/929552).

### Ethics statement

GenV obtained ethical and governance approval from the Royal Children’s Hospital Human Research Ethics Committee (HREC/69151/RCHM-2020) for the microbiome analysis of anonymised residual clinical GBS screening swabs transferred from Austin Pathology, Victoria, Australia in phase one, and the collection and analysis of vaginal/vaginal-rectal swabs for research from Sunshine Hospital, Victoria, Australia in phase two of the study, with additional governance approval from Western Health. Consent for the use of leftover clinical specimens in phase one was waived by the Royal Children’s Hospital Human Research Ethics Committee (HREC 2019.011) according to the National Health and Medical Research Council (NHMRC) National Statement (Section 2.3.10). Informed written consent was obtained from research participants prior to the collection of research swabs in phase two. All methods were performed in accordance with the relevant guidelines and regulations.

### Role of funders

The funders were not involved in study design, data collection, analysis, interpretation, or writing.

## Results

### Phase I: Residual clinical vaginal/vaginal-rectal swabs: assessment of microbial diversity

In phase I, we analysed 20 samples out of the 64 residual clinical GBS swabs from the pathology provider. The residual volume in the clinical swabs was relatively high with 89% (57/64) samples containing ≥ 750 µL and 98% (63/64) of samples containing ≥ 700 µL (the remaining sample contained 650 µL). Bacterial DNA was extracted and amplified from all 20 residual clinical swabs tested, with DNA concentrations ranging between 5·5 and 206·6 ng/µL (median 37·5 ng/µL; IQR 23–84 ng/µL). Following 16S PCR amplification, all samples produced a visible PCR product of the expected size when visualized by gel electrophoresis (Supplementary Figure S1). All negative controls had no detectable product and failed quality control during sequencing library preparation due to very low DNA concentrations.

**Figure 2:**
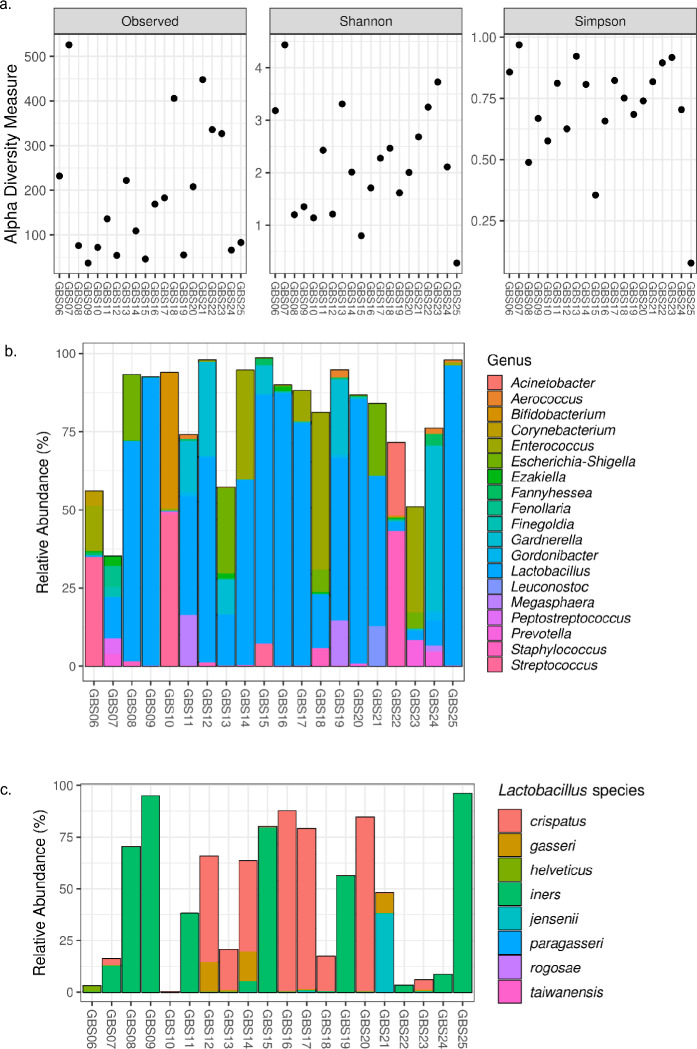
Microbial diversity and composition of pathology-provided GBS swabs. Diversity (**a**) measured by richness (observed ASVs), Shannon diversity index and Simpson diversity index. Relative abundances (%) are shown for the top 50 most common ASVs to genus level taxonomy (**b**) and the *Lactobacillus* genus by species (**c**). In figure (**b**), individual ASVs belonging to the same genus are grouped together.

Following sequencing and analysis of the 16S products, a range of microbial diversity measures were derived across the 20 samples (Fig. 2a). Richness varied from 37 to 526 (median 153 ASVs; IQR 68- 303 ASVs). The diversity also varied greatly across the samples. The Shannon diversity index ranged from 0·28 to 4·4 (median 2·06; IQR 1·25–3·06) and Simpson diversity index (representing a probability that ranges from 0 to 1) ranged from 0·08 to 0·97 (median 0·75; IQR 0·63–0·85).

*Lactobacillus* was the predominant genus in 10/20 (50%) samples (> 50% relative abundance) and was present to a lesser degree in all other samples (Fig. 2b). *Streptococcus agalactiae* (GBS) had a relative abundance higher than 25% in 3/20 (15%) samples. The dominant *Lactobacillus* species was typically either *Lactobacillus crispatus* or *Lactobacillus iners* (Fig. 2c).

### Phase II: Dedicated GBS research swabs: assessment of time and temperature on diversity measures

The 20 vaginal/vaginal-rectal swabs in Amies media were received for processing within four hours of collection and were kept at either 4 °C or at room temperature. Aliquots of refrigerated samples were taken at day 0, 1, 5, and 10, and aliquots of room temperature samples were taken at 0, 4, 24, and 48 h. Bacterial DNA was successfully extracted and amplified from all samples, however the day 1 aliquot from sample SH15 failed sequencing quality control for unknown reasons (all the blank controls also failed sequencing quality control).

#### Samples stored at 4 °C

**Figure 3:**
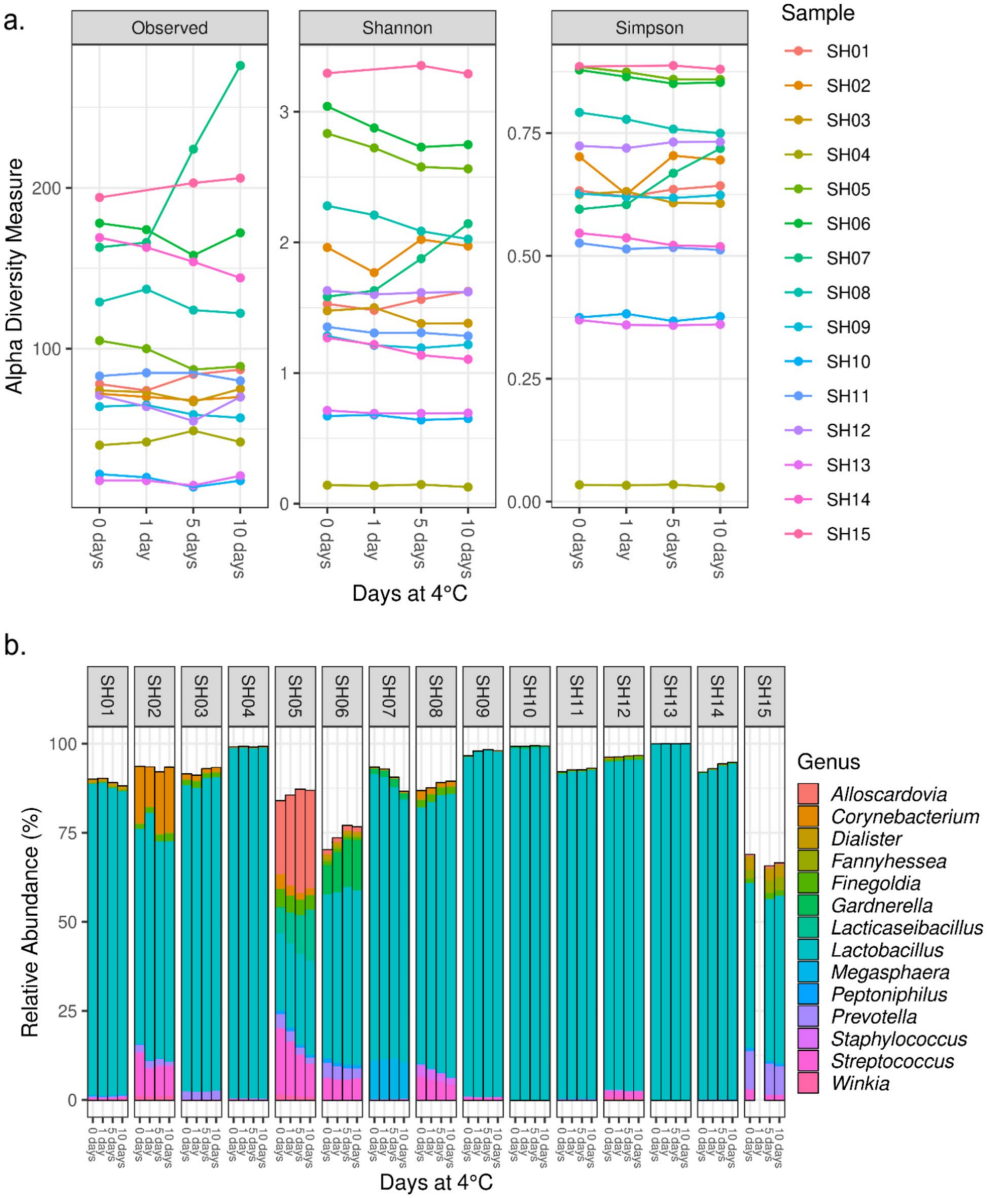
Microbial diversity and composition of dedicated GBS swabs stored at 4 °C. Swabs were sampled on collection (0 days) and stored at 4 °C with additional sampling on day 1, day 5 and day 10. Diversity (**a**) measured by richness (observed ASVs), Shannon diversity index and Simpson diversity index. Relative abundances (%) are shown for the top 50 most common ASVs to genus level taxonomy (**b**) for each sample at each time point. In figure (**b**), individual ASVs belonging to the same genus are grouped together.

When stored at 4 °C, richness and diversity remained relatively stable over the ten day period (Fig. 3a). Only one sample (SH07) had significant changes in richness and diversity over the ten day period (richness increased from 163 on day 0 to 274 on day 10). Across all samples, the median difference from day 0 to day 10 was 2 (median 78; IQR 67.5–146 to median 80; IQR 63.5–133, respectively), 0·09 (median 1.53; IQR 1.27–2.28 to median 1.62 and IQR 1.11–2.14, respectively), and 0·01 (median 0.63; IQR 0.54–0.76 to median 0.64; IQR 0.52–0.74, respectively) for richness, Shannon diversity, and Simpson diversity, respectively. In terms of composition, *Lactobacillus* was present in all samples and was the dominant genus (> 50% relative abundance) in 12/15 (80%) of samples at day 0 (Fig. 3b). No other genera were dominant in these samples, however *Alloscardovia*, *Streptococcus*, *Lacticaseibacillus*, *Corynebacterium* and *Gardnerella* were all present at a relative abundance > 15% in at least one sample across all timepoints.

The microbiota composition remained relatively stable across the ten days at 4 °C (Supplementary Table S1 and Figures S2-S6 for beta-diversity). Only two of the samples had a change in relative abundance more than ten percentage points. The relative abundance of *L. iners* (ASV1) in sample SH07 decreased from 61·2% to 48·2% and the relative abundance of *L. gasseri* (ASV23) in sample SH12 increased from 23·17% to 33·87%. However, for most samples (11/15, 73%), the change in relative abundance across all ASVs was less than five percentage points, and for three samples (3/15, 20%) the change in relative abundance across all ASVs was less than one percentage point. We also explored whether there were significant differences in composition detected on the day of collection (day 0) compared to day 10 using DESeq2, finding that five ASVs were significantly higher and five were significantly lower after 10 days (Supplementary Figure S7 and Supplementary Table S2). However, except for *L. jensenii*, all changes were for ASVs present in samples at less than 1% relative abundance and therefore unlikely to be a biologically meaningful change (Supplementary Figure S8). In sample SH07, *L. jensenii* had a higher relative abundance on day 0 (9·09%) compared to day 10 (17·95%).

#### Samples stored at room temperature

**Figure 4:**
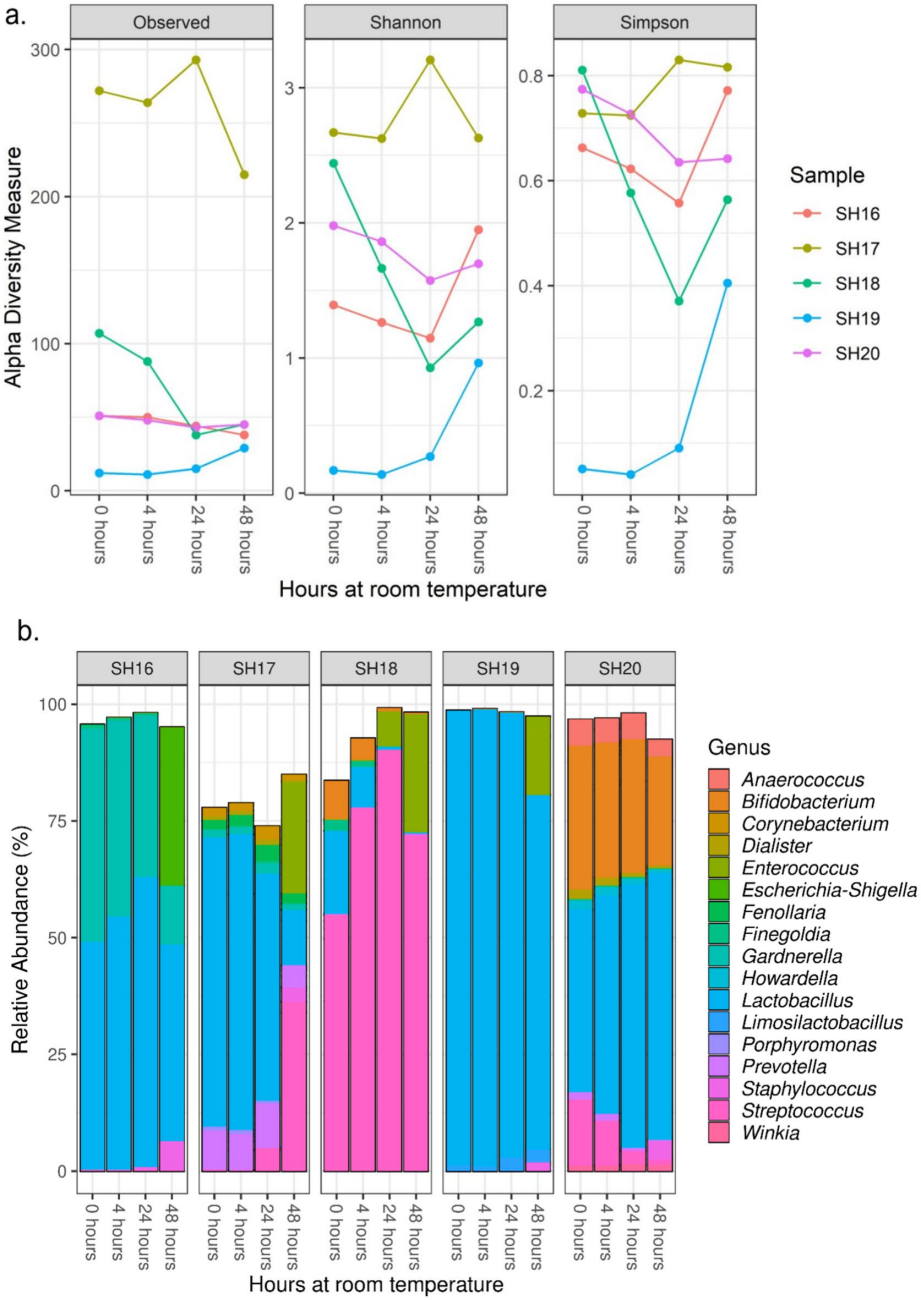
Microbial diversity and composition of dedicated GBS swabs stored at room temperature. Swabs were sampled on collection (0 h) and stored at room temperature with additional sampling after 4 h, 24 h and 48 h. Diversity (**a**) measured by richness (observed ASVs), Shannon diversity index and Simpson diversity index. Relative abundance (%) is shown for the top 50 most common ASVs to genus level taxonomy (**b**) for each sample at each time point. In figure (b), individual ASVs belonging to the same genus are grouped together.

Storage at room temperature had some impact on richness and diversity (Fig. 4a) (see Supplementary Table S1 and Figures S2-S6 for beta-diversity). Across the five samples, the median difference from 0 to 48 h was 6 (median 51; IQR 51–107 to median 45; IQR 38–45, respectively), 0·28 (median 1.98; IQR 1.39–2.44 to median 1.70; IQR 1.27–1.95, respectively), and 0·09 (median 0.73; IQR 0.66–0.77 to median 0.64; IQR 0.56–0.77, respectively) for richness, Shannon diversity, and Simpson diversity, respectively. The starting composition of samples kept at room temperature was dominated by *Lactobacillus* (> 50% relative abundance) in two out of the five samples (40%) and *Streptococcus* was dominant in one of the five samples (20%) (Fig. 4b). No other genus was dominant, however *Gardnerella* and *Bifidobacterium* were present at a relative abundance above 30% in the starting composition of at least one sample each. For all five samples stored at room temperature, the microbiota composition shifted over 48 h so that the sample was no longer broadly representative of the fresh sample. In contrast, few changes were seen after only four hours at room temperature. One sample (SH18) had a change in relative abundance of more than 10 percentage points—GBS (ASV8) increased from 36·33% to 63·90% relative abundance. Moderate changes were seen after 24 h at room temperature, with four out of five samples having a change in relative abundance of more than 10 percentage points in at least one ASV. After 48 h at room temperature a change in the relative abundance of more than 10 percentage points occurred in at least two ASVs for all five samples. The most extreme case was a decrease in the relative abundance of *L. iners* (ASV1) from 50·47% at 0 h to 9·49% at 48 h in sample SH17.

**Figure 5:**
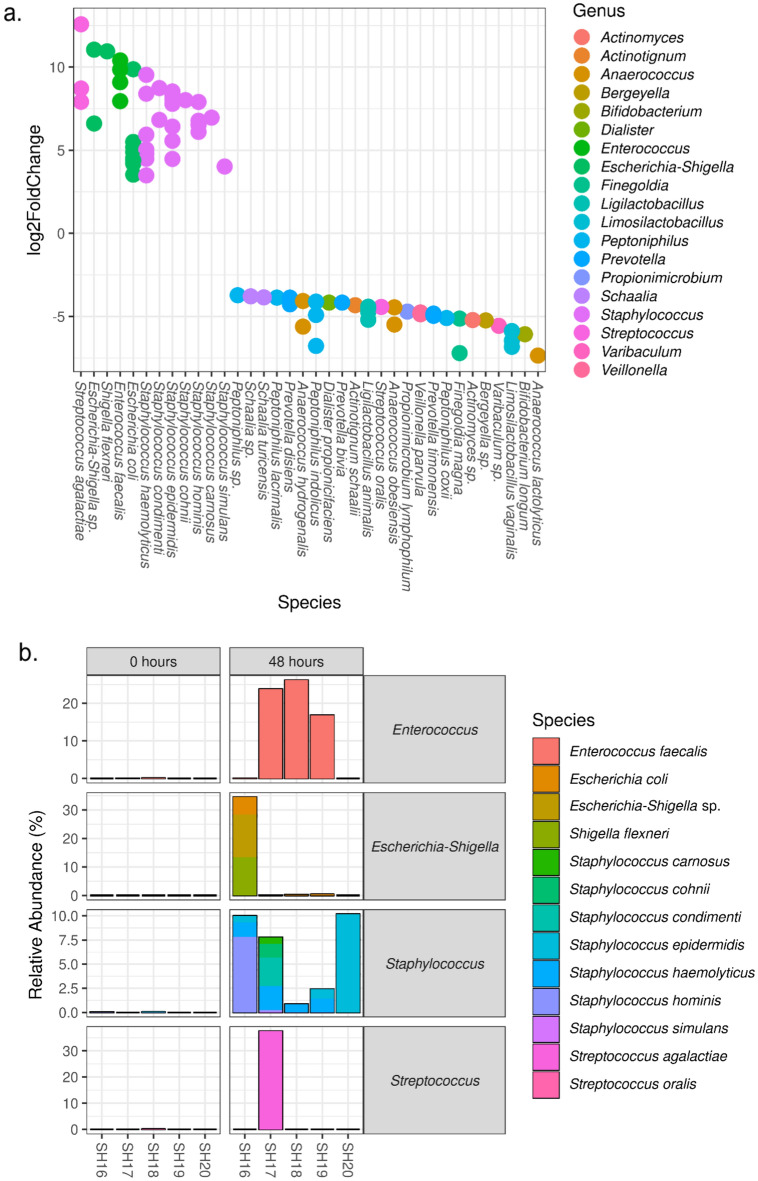
ASVs that were significantly different between 0 and 48 h for dedicated GBS swabs stored at room temperature (adjusted p-value < 0.05). DESeq2 analysis of differential relative abundance between 0 and 48 h at room temperature (**a**). For significantly different ASVs, the relative abundance (%) for genera with a relative abundance over 2.5% are shown (**b**).

We examined the differential relative abundance of microbiota at 0 h compared with 4 h using DESeq2 and saw no changes. We noted some smaller changes in differential relative abundance in microbiota after 24 h at room temperature (Supplementary Figure S9 and Supplementary Table S3). When we examined the differential relative abundance of microbiota at 0 h compared with 48 h (Supplementary Table S4), we found significant increases for ASVs from the genera *Streptococcus* (n = 3), *Escherichia*-*Shigella* (n = 11), *Enterococcus* (n = 4), and *Staphylococcus* (n = 24) (Fig. 5a). In addition to the statistical significance, these represented likely biologically significant changes in composition (Fig. 5b). In all the five samples, these ASVs were present at a very low relative abundance (< 0.15%) on receipt of the sample, however over the course of 48 h at room temperature each of these genera increased to 10–30% relative abundance in at least one sample.

We also explored whether sample site could be inferred based on differences in the dedicated vaginal/vaginal-rectal swabs. Using random forest analysis, the most important ASV for predicting sample site was *L. jensenii* (ASV10), followed by *Gardnerella vaginalis* (ASV27), both of which were more abundant in vaginal-rectal samples compared with vaginal-only samples (Supplementary Figure S10).

## Discussion

The potential for residual clinical samples for measuring the pregnancy microbiome has yet to be fully explored. Here, we assessed the suitability of residual clinical swabs after GBS screening in Liquid Amies for profiling the vaginal-rectal maternal microbiome in late pregnancy. Firstly, we tested whether the post-GBS testing sample was of use for the generation of a diverse bacterial microbiome and then we tested the impact of storage temperature and time post-collection on the microbial profile.

We successfully amplified bacterial DNA from all 20 samples and found that compositionally, the microbiota found were in line with earlier studies of vaginal microbiota. Most of our repurposed pathology samples were dominated by previously described CSTs with the majority dominated by either *L. iners* (CST-III) or *L. crispatus* (CST-I), and one sample dominated by *L. jensenii* (CST-V)^5,6^. Highlighting the utility of residual clinical GBS swabs, all samples we received contained enough material for multiple DNA extractions to generate microbiome data which can be done confidently with just over 200 µL of swab media.

We next assessed the impact of common storage conditions on the microbiota profiles using dedicated research swabs collected specifically for this study. With many of the clinical residual swabs having been stored for up to ten days at 4 °C, we explored whether this would have a significant impact on the observed microbiota. Our results show that while some changes were seen over the ten days at 4 °C, these were minimal with only one ASV (*L. jensenii*) having a biologically significant change over this period. It would be expected that some minor variability in microbiota profiles would occur by sampling variability alone, regardless of storage condition. The results after ten days were broadly representative of the results on the day of collection.

Perhaps unsurprisingly, our results show that after 48 h at room temperature, swabs can no longer be considered representative of freshly collected swabs. The major shifts in composition were specifically in *Enterococcus faecalis*, *Escherichia*-*Shigella* sp., *Staphylococcus* sp. and GBS. The significant increase in the relative abundance of GBS in sample SH017 from 0.11% to 37.54% resulted in this being the most common taxa in the sample rather than *L. iners* which was most common at sample collection (61.95%). If microbiota composition were examined for samples under these conditions, misleading conclusions may be drawn about community profiles and associations between microbiota and with disease states.

Some changes were also seen in composition and diversity after 24 h at room temperature. Unlike differences seen at 48 h, changes in sample composition and diversity were less marked and not universal across all five samples. This suggests that the initial composition of the sample may influence the relative impact of room temperature on sample composition. Few studies have examined differences in storage conditions for the vaginal-rectal microbiome. Bai et al. found that although no significant differences were found between common storage conditions (− 20 °C and – 80 ˚C) overall, vaginal samples with greater starting diversity (CST-IV) were most impacted by differing storage conditions^11^. Although being a vastly different sample type, the human gut microbiome has been extensively studied and findings from faecal samples suggest the relative impact of storage conditions may also be dependent on starting composition given the inconsistent reports on the impact of room temperature storage^26–28^.

While it was expected that these swabs would contain a microbiome consistent with the vaginal microbiome, owing to the dominance of *Lactobacillus* species, the contribution of microbiota from the rectal sampling was less obvious. One of the key limitations in repurposing vaginal/vaginal-rectal swabs collected by pathology providers is the lack of definitive data on how each swab was sampled including consistency with collection instructions. Although vaginal-only sampling has been shown to reduce sensitivity of GBS testing compared with vaginal-rectal sampling, there is variability in collection practices^29,30^. This was also clear for our dedicated research swabs, largely patient-collected, where approximately two-thirds of samples appear vaginal-only. We carried out a preliminary statistical analysis aimed at discriminating sampling site based on microbial composition, but this was limited by the small sample size. The most important ASV in the random forest model we performed, *L. jensenii*, appeared to have a higher relative abundance in vaginal-rectal samples compared with vaginal-only samples. However, it was still present in vaginal-only samples and this result may not hold true with a larger dataset. Similarities between vaginal and rectal microbiota for other species have been seen at both a species and strain level in other studies which may complicate the discernment of sampling sites^31,32^.

Earlier studies have suggested that the sample collection site/s will affect the microbial detection rate from vaginal/vaginal-rectal swabs^10,33^. While the combined vaginal-rectal swab has a higher microbial detection sensitivity than vaginal swab or rectal swab alone^29,33^, a comparison between patient- versus physician-collected swabs showed a high similarity, with no significant changes in alpha diversity^34^. Thus, self-collection appears to be a valid sampling approach to assess the late pregnancy maternal microbiome. In conjunction with results from this pilot study, we propose that residual swab transport media, collected for GBS pregnancy screening, represents a valid sample for generating late pregnancy microbiome profiles.

It is worth noting that we only tested recovery of microbiota from swabs liquid Amies media. GBS swabs are also often transported in Amies gel agar media, which may yield different results and may not be appropriate for large scale studies. The small sample size in this pilot study limited some analyses. This was true particularly for samples stored at room temperature, however despite the small number we were still able to show the negative impact of room temperature storage for 48 h. Although relatively small in scale, this proof-of-concept study highlights the research utility of residual clinical GBS swabs stored at 4 °C for microbiome research, including in large population-based cohorts studies. Our data suggest little need for freezing at − 30 °C or − 80 °C to maintain integrity of the sampled microbiome, though this needs to be tested directly. Future studies could expand on this pilot study and examine the effects of storage conditions on a larger scale. Studies could further investigate a range of GBS swab conditions and other storage media, including longer-term storage of frozen samples (at − 20 °C and − 80 °C) to better understand if samples remain stable in the swab media and/or potential effects of freeze-thawing. The impact of freezing at longer periods of time (for years) would be particularly relevant in assessing the utility of large scale biobanking of such swab material.

In conjunction with results from this pilot study, we propose that residual swab transport media, collected for GBS pregnancy screening, and stored at 4 °C represents a valid sample for generating late pregnancy microbiome profiles. These results confirm the collection and biobanking of residual clinical GBS swabs for large life course studies like GenV and open possibilities for their use to address many unanswered research questions.

### Supplementary Information

Supplementary Information.

## Data Availability

The dataset generated and analysed in the current study is available in the NCBI Sequence Read Archive, accession number PRJNA929552 (http://www.ncbi.nlm.nih.gov/bioproject/929,552). All the data associated with this study are presented in the paper or the Supplementary Materials. Additional information needed to reanalyse the data reported in this paper is available from the corresponding author upon reasonable request.
