# Supplementary material for: The potential of residual clinical Group B Streptococcus swabs for assessing the vaginorectal microbiome in late pregnancy
Source: Sci Rep. 2024 Aug 20;14:19318. doi: 10.1038/s41598-024-70431-5 (PMC11336092; doi:10.1038/s41598-024-70431-5)
Supplement: Supplementary file 1 — Supplementary Information. [file 41598_2024_70431_MOESM1_ESM.docx]

**Supplemental material**


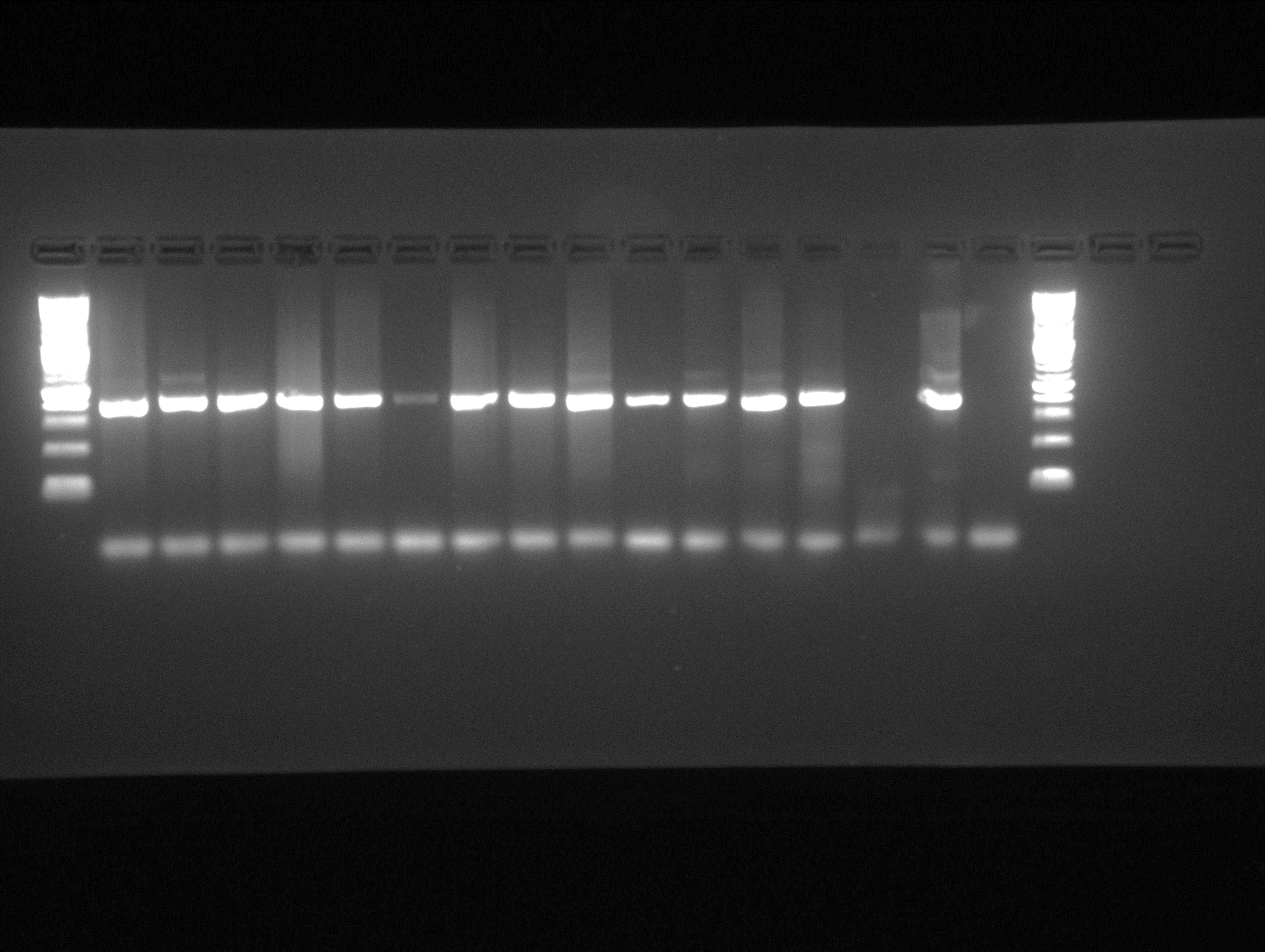

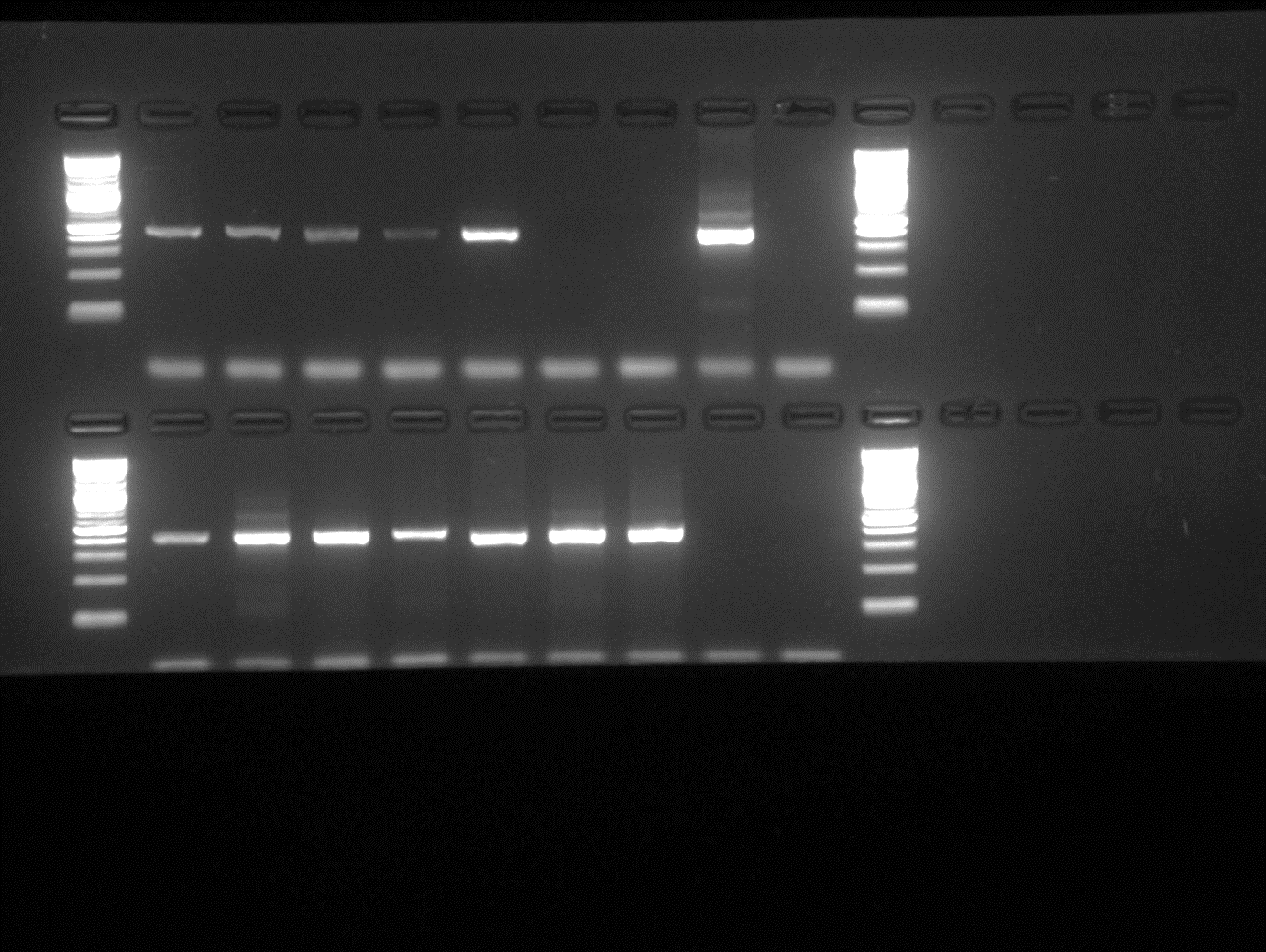


Positive control

PCR no template

Extraction reagents

Extraction reagents

Liquid Amies media

GBS12

GBS11

GBS10

GBS09

GBS07

GBS06

GBS08

GBS14

GBS13

GBS25

GBS24

GBS23

GBS22

GBS21

GBS20

GBS19

GBS18

GBS17

GBS16

GBS15

**Figure S1. 16S rRNA gene PCR products on 2% w/v agarose gels.** PCR products are labelled above wells and include DNA extracted from excess clinical vaginal/vaginal-rectal swabs (GBS06-GBS25), a blank liquid Amies media control and reagent-only extraction controls, as well as a positive control and a PCR no template control. PCR products were run alongside a 1 kb Plus DNA ladder (New England Biolabs).

**Table S1. Summary of statistical analysis of alpha- and beta-diversity measures for each storage condition of phase II.**

| **Storage condition** | **Measure** | **Statistical test** | **p-value** |
| --- | --- | --- | --- |
| 4°C  (n=15) |  |  |  |
|  | **Alpha-diversity** |  |  |
|  | Richness | Wilcoxon signed-rank (0 to 10 days) | 0.532 |
|  | Shannon diversity | Wilcoxon signed-rank (0 to 10 days) | 0.064 |
|  | Simpson diversity | Wilcoxon signed-rank (0 to 10 days) | 0.095 |
|  | **Beta-diversity** |  |  |
|  | unweighted UniFrac | PERMANOVA | 1.000 |
|  | weighted UniFrac | PERMANOVA | 1.000 |
|  | Bray-Curtis | PERMANOVA | 1.000 |
|  | Jaccard | PERMANOVA | 1.000 |
| Room temperature  (n=5) |  |  |  |
|  | **Alpha-diversity** |  |  |
|  | Richness | Wilcoxon signed-rank (0 to 48 hours) | 0.313 |
|  | Shannon diversity | Wilcoxon signed-rank (0 to 48 hours) | 1.000 |
|  | Simpson diversity | Wilcoxon signed-rank (0 to 48 hours) | 1.000 |
|  | **Beta-diversity** |  |  |
|  | unweighted UniFrac | PERMANOVA | 0.986 |
|  | weighted UniFrac | PERMANOVA | 0.903 |
|  | Bray-Curtis | PERMANOVA | 0.997 |
|  | Jaccard | PERMANOVA | 0.999 |


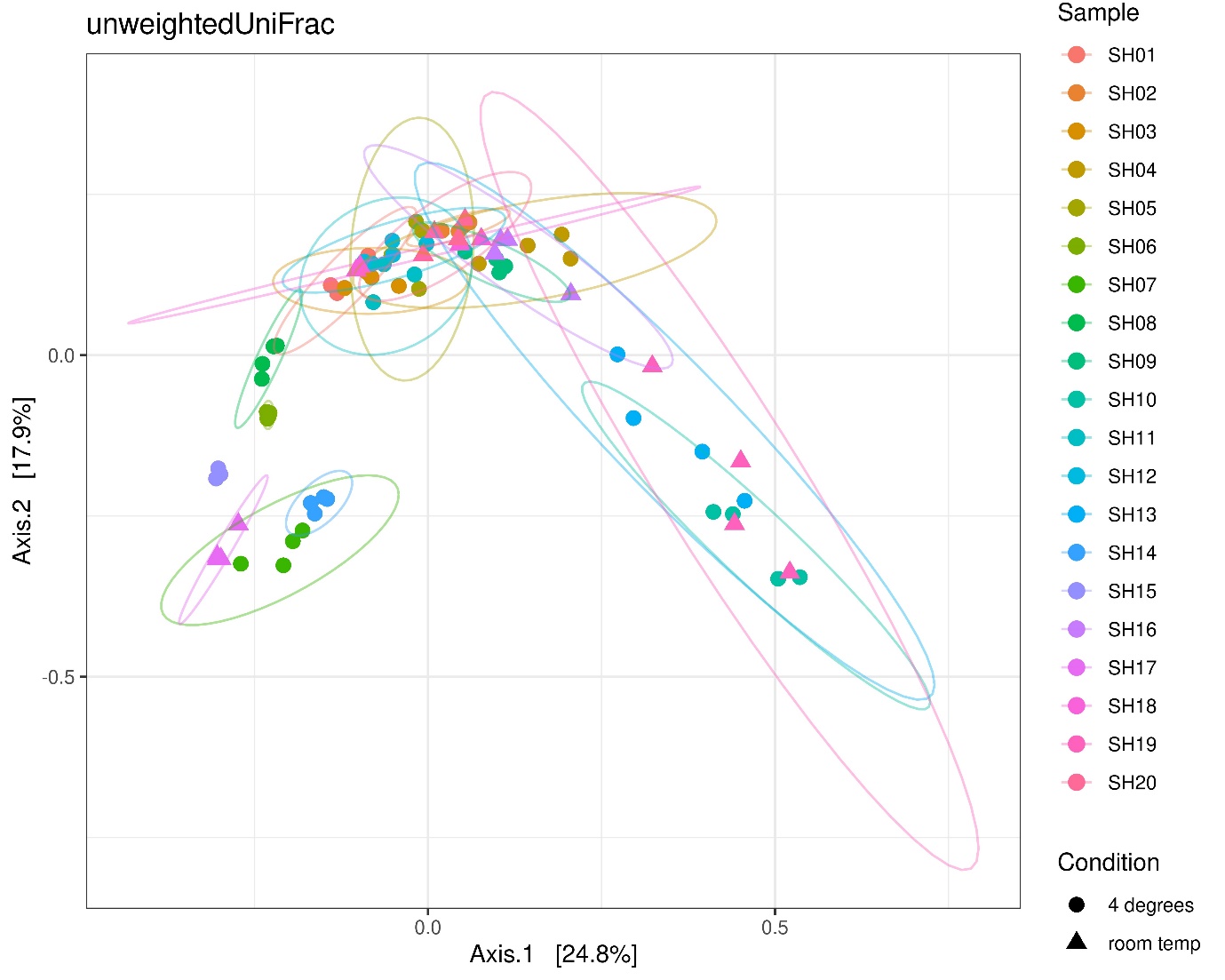


**Figure S2. An unweighted UniFrac Principle Coordinate Analysis (PCoA) plot of microbial community composition at each timepoint for all phase II samples.** Sample stored at 4°C are shown as circle and samples at room temperature are shown as triangles. Ellipses represent the 95% confidence level for a multivariate normal distribution.


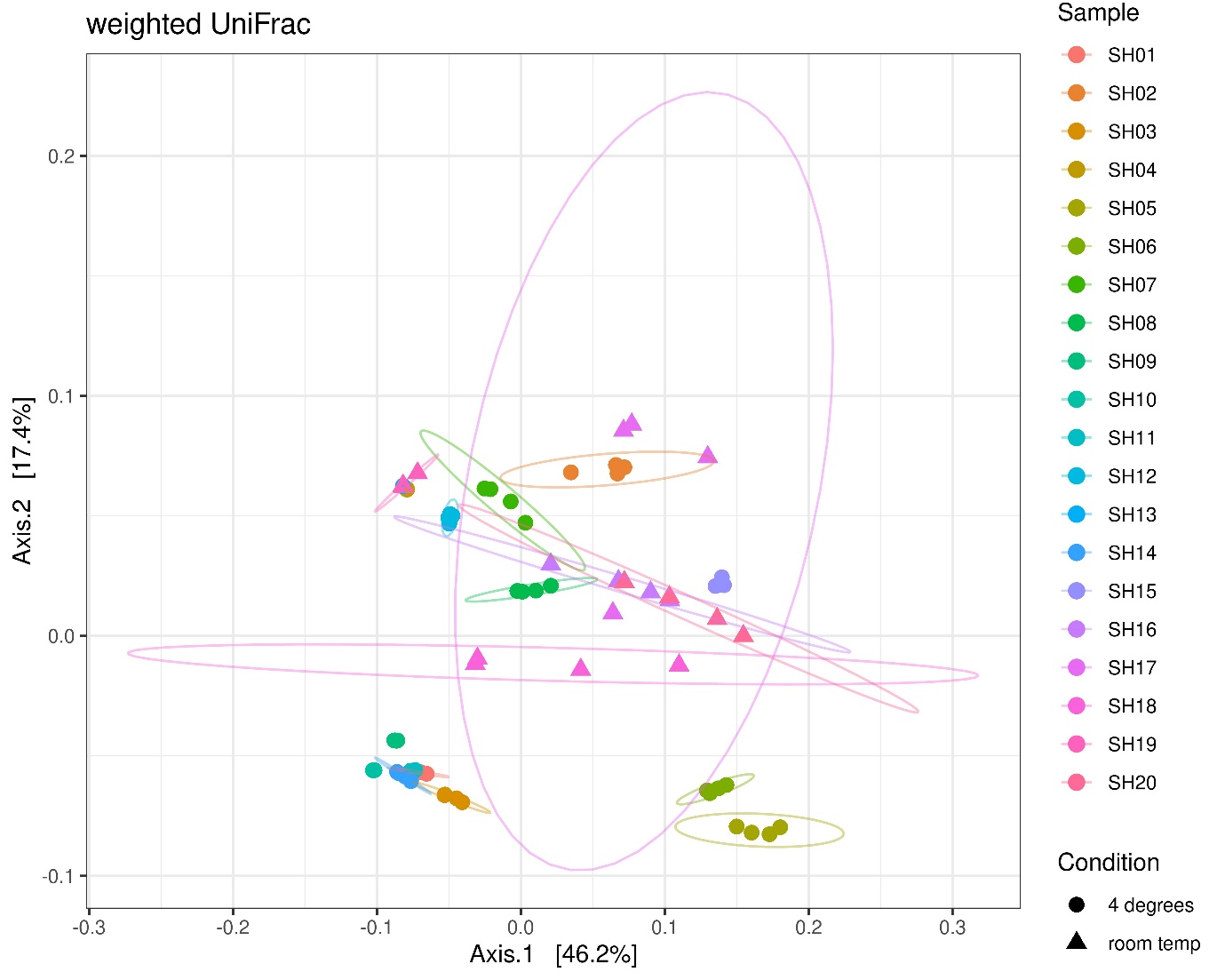


**Figure S3. A weighted UniFrac PCoA plot of microbial community composition at each timepoint for all phase II samples.** Sample stored at 4°C are shown as circles and samples at room temperature are shown as triangles. Ellipses represent the 95% confidence level for a multivariate normal distribution.


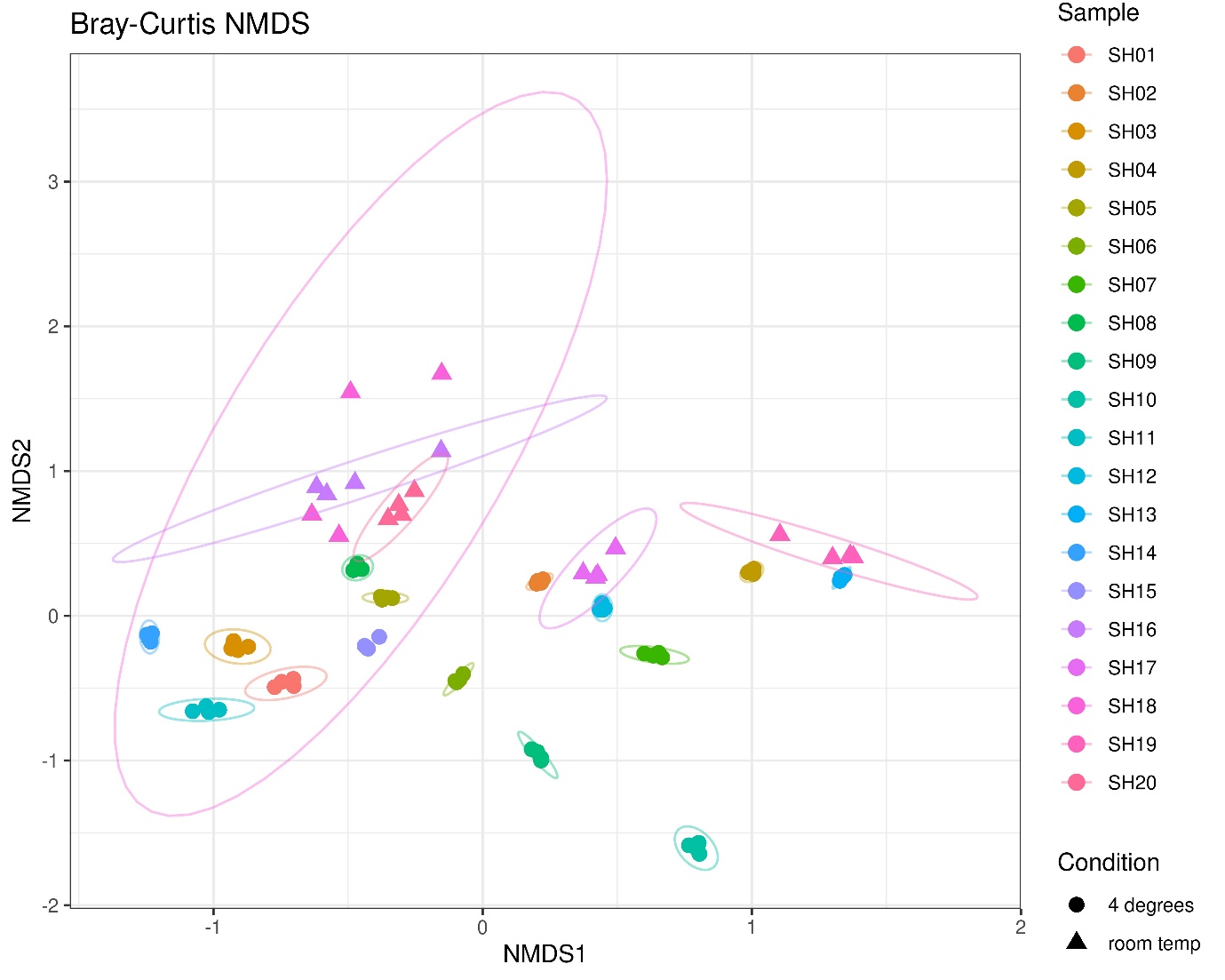


**Figure S4. A non-metric multidimensional scaling (nMDS) plot of microbial community composition at each timepoint for all phase II samples.** Distances were calculated using the Bray-Curtis method. Sample stored at 4°C are shown as circles and samples at room temperature are shown as triangles. Ellipses represent the 95% confidence level for a multivariate normal distribution.


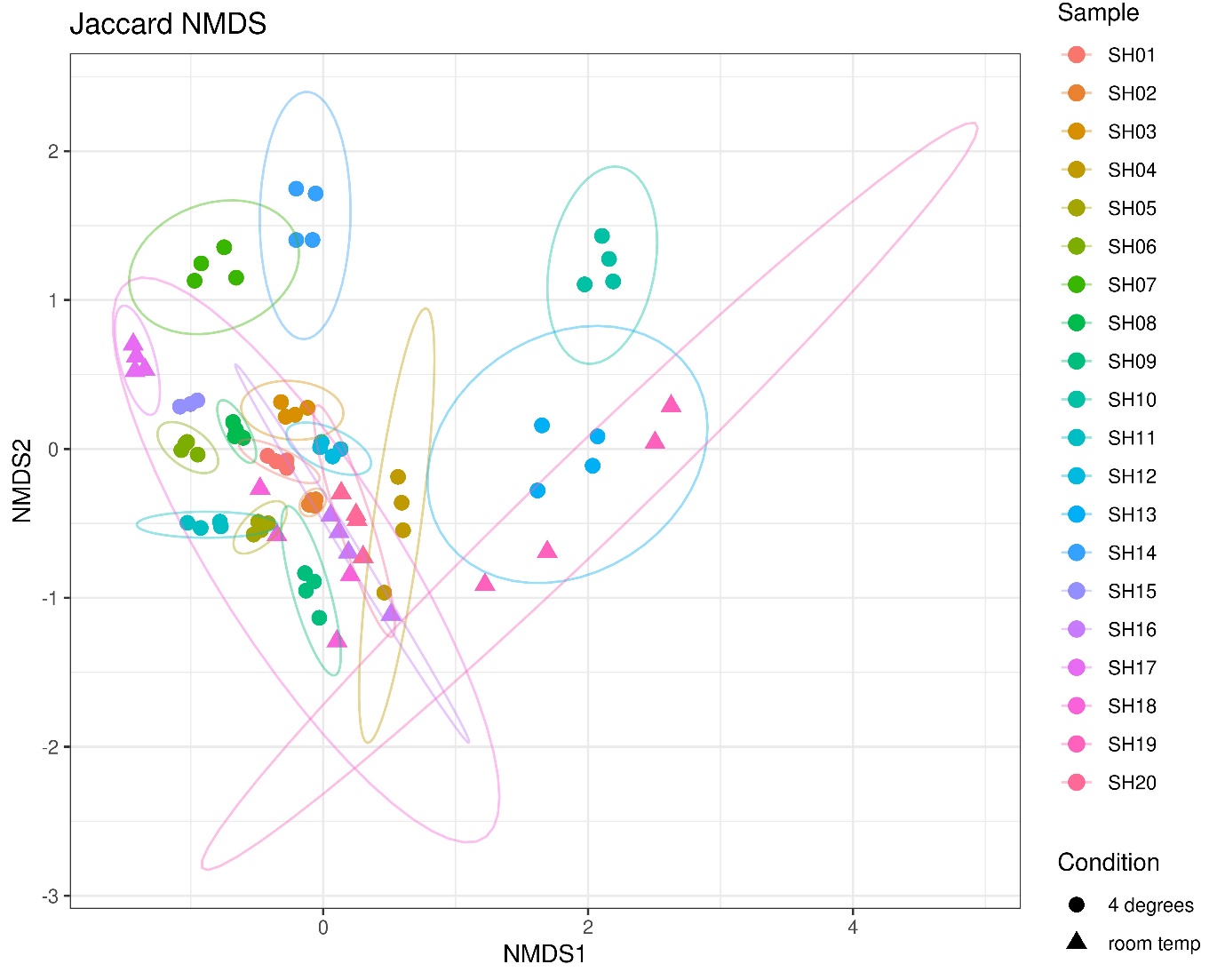


**Figure S5. A nMDS plot of microbial community composition at each timepoint for all phase II samples.** Distances were calculated using the Jaccard method. Sample stored at 4°C are shown as circles and samples at room temperature are shown as triangles. Ellipses represent the 95% confidence level for a multivariate normal distribution.


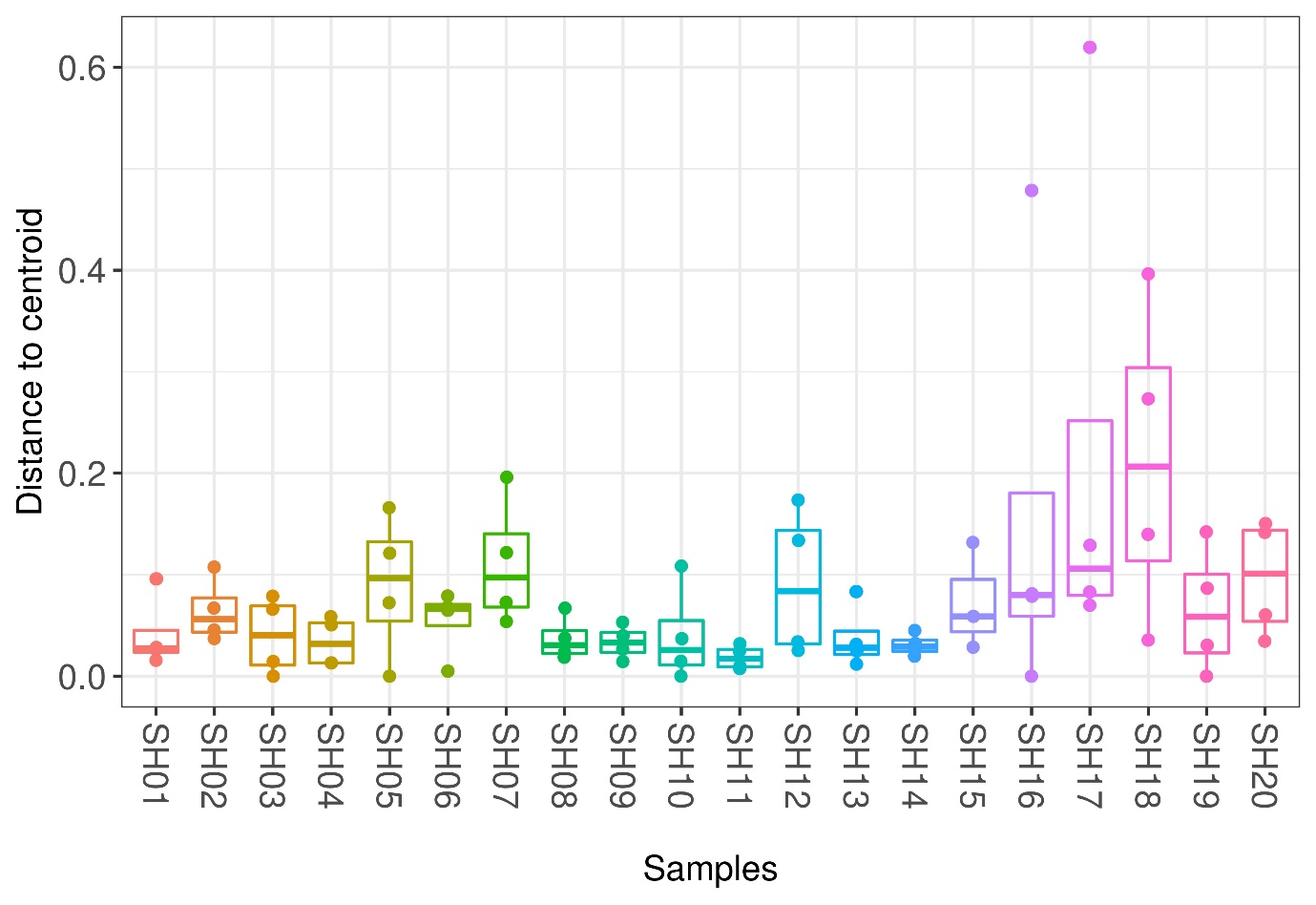


**Figure S6. Dispersion of community composition for each sample across timepoints for all phase II samples.** Distances were calculated using the Bray-Curtis method. Box plots are median and interquartile range. Samples SH01-SH15 were stored at 4°C, and samples SH16-SH20 were stored at room temperature. Closely grouped timepoints are indicative of closely matched microbial composition within a sample.


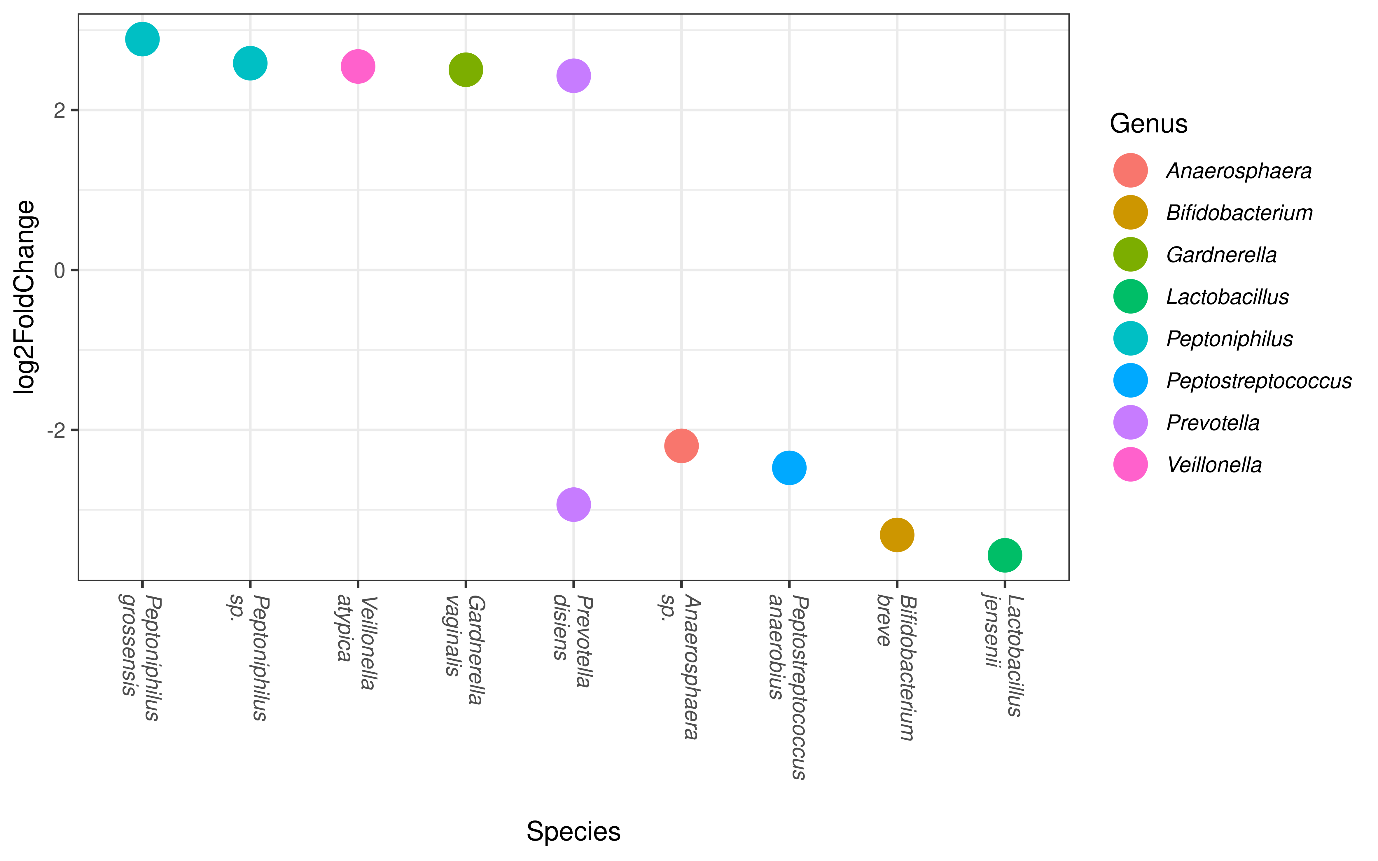


**Figure S7. Differential relative abundance analysis (using DESeq2) of composition at 10 days compared with the day of collection (day 0).**

**Table S2. Differential relative abundance analysis (using DESeq2) of composition at 10 days compared with the day of collection (day 0)**

| **ASV** | **Base**  **Mean** | **log2**  **Fold**  **Change** | **p-value** | **adjusted p-value** | **Species** |
| --- | --- | --- | --- | --- | --- |
| ASV10 | 290.6 | -3.57 | 0.001 | 0.033 | *Lactobacillus jensenii* |
| ASV17 | 6.5 | -3.31 | <0.001 | 0.019 | *Bifidobacterium breve* |
| ASV46 | 3.6 | -2.47 | 0.001 | 0.029 | *Peptostreptococcus anaerobius* |
| ASV130 | 5.0 | 2.59 | 0.001 | 0.029 | *Peptoniphilus* sp. |
| ASV333 | 11.7 | 2.89 | <0.001 | 0.029 | *Peptoniphilus grossensis* |
| ASV391 | 4.1 | 2.54 | 0.001 | 0.029 | *Veillonella atypica* |
| ASV506 | 3.9 | 2.50 | 0.001 | 0.033 | *Gardnerella vaginalis* |
| ASV554 | 3.8 | 2.43 | 0.001 | 0.033 | *Prevotella disiens* |
| ASV681 | 4.8 | -2.93 | <0.001 | 0.020 | *Prevotella disiens* |
| ASV697 | 3.3 | -2.20 | 0.002 | 0.039 | *Anaerosphaera* sp. |


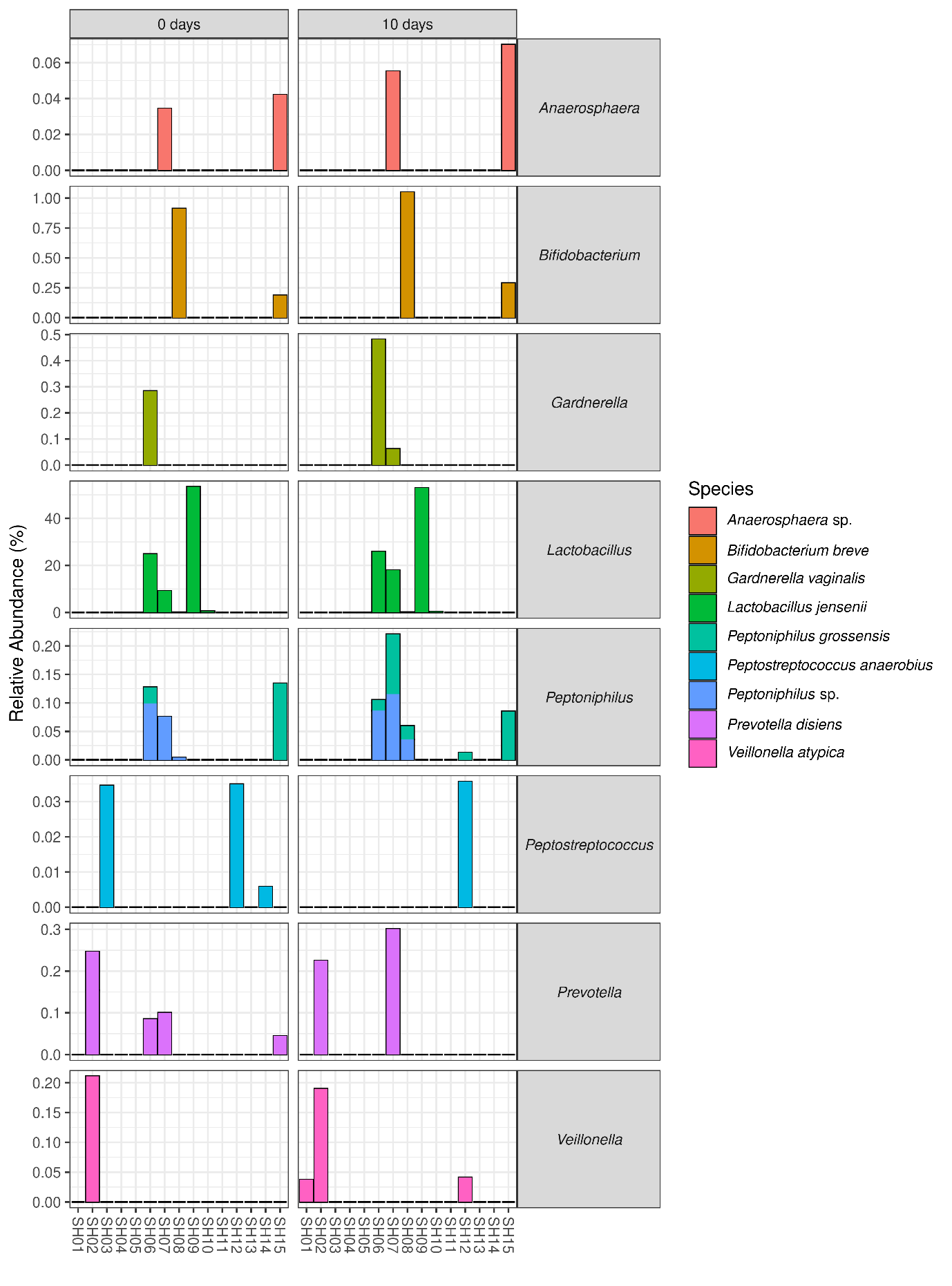


**Figure S8. Relative abundance (%) of ASVs that were significantly different after 10 days at 4°C compared with day 0.** Differential relative abundance analysis was done using DESeq2.


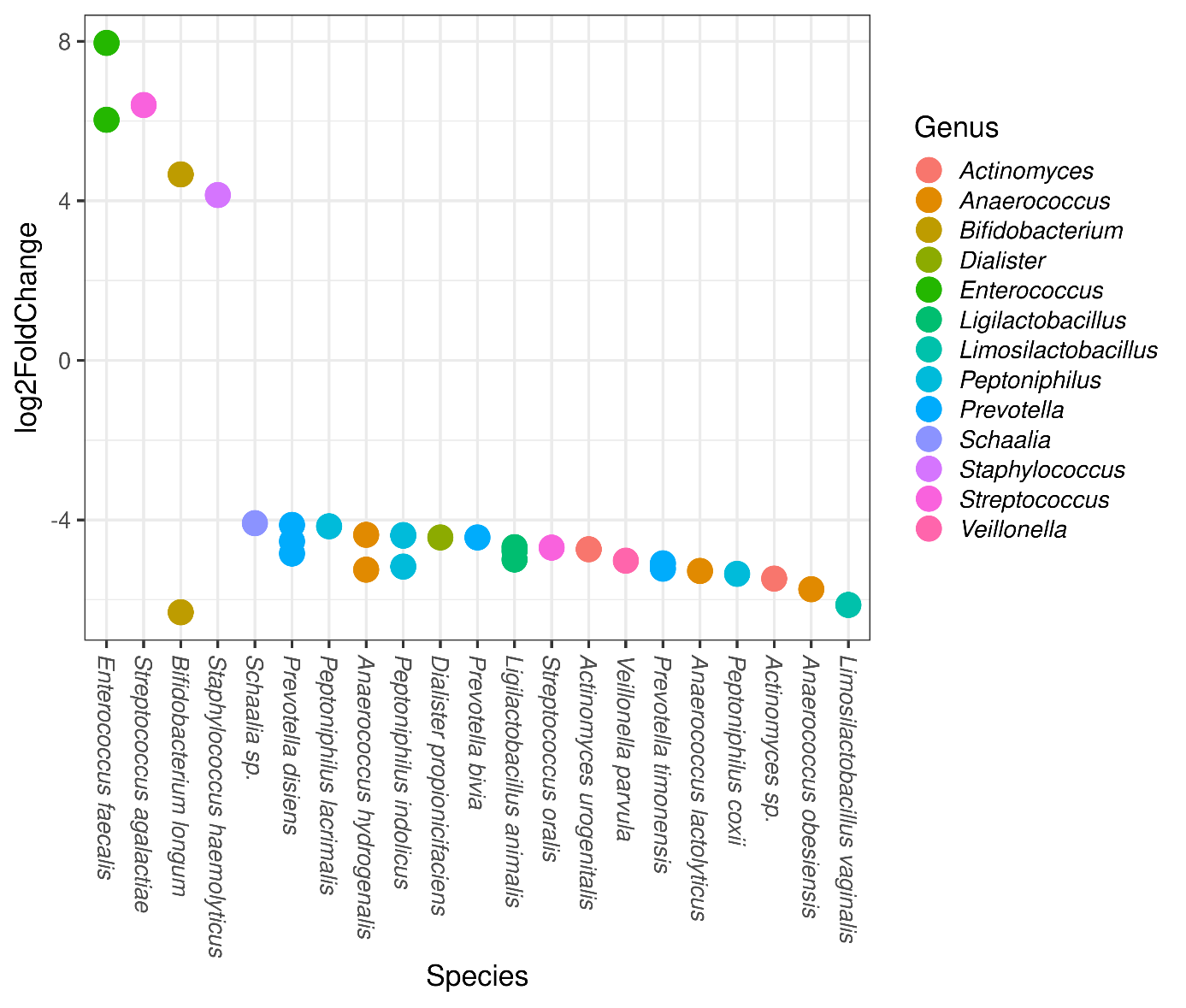


**Figure S9.** **Differential relative abundance analysis (using DESeq2) of composition for 24 hours at room temperature compared with on receipt of sample.**

**Table S3. Differential relative abundance analysis (using DESeq2) of composition for 24 hours at room temperature compared with on receipt of sample.**

| **ASV** | **Base**  **Mean** | **log2**  **Fold**  **Change** | **p-value** | **adjusted p-value** | **Species** |
| --- | --- | --- | --- | --- | --- |
| ASV11 | 54.0 | 6.40 | <0.001 | 0.034 | *Streptococcus agalactiae* |
| ASV35 | 42.2 | 6.03 | 0.001 | 0.034 | *Enterococcus faecalis* |
| ASV75 | 11.6 | 4.14 | 0.004 | 0.045 | *Staphylococcus haemolyticus* |
| ASV106 | 12.0 | -4.16 | 0.006 | 0.049 | *Peptoniphilus lacrimalis* |
| ASV134 | 14.1 | -4.39 | 0.006 | 0.049 | *Peptoniphilus indolicus* |
| ASV197 | 19.8 | -4.12 | 0.006 | 0.049 | *Prevotella disiens* |
| ASV207 | 91.4 | -5.28 | 0.004 | 0.045 | *Anaerococcus lactolyticus* |
| ASV226 | 160.2 | 7.96 | <0.001 | 0.018 | *Enterococcus faecalis* |
| ASV233 | 23.8 | -5.17 | 0.002 | 0.043 | *Peptoniphilus indolicus* |
| ASV293 | 38.6 | -5.25 | 0.002 | 0.043 | *Anaerococcus hydrogenalis* |
| ASV349 | 35.1 | -5.74 | 0.001 | 0.042 | *Anaerococcus obesiensis* |
| ASV358 | 14.5 | -4.44 | 0.006 | 0.049 | *Dialister propionicifaciens* |
| ASV386 | 11.4 | -4.08 | 0.007 | 0.049 | *Schaalia* sp. |
| ASV488 | 21.5 | -5.02 | 0.003 | 0.043 | *Veillonella parvula* |
| ASV587 | 19.0 | -4.84 | 0.003 | 0.043 | *Prevotella disiens* |
| ASV739 | 27.0 | -5.35 | 0.002 | 0.043 | *Peptoniphilus coxii* |
| ASV777 | 52.3 | -6.31 | 0.001 | 0.034 | *Bifidobacterium longum* |
| ASV803 | 17.6 | -4.74 | 0.003 | 0.043 | *Actinomyces urogenitalis* |
| ASV931 | 46.1 | -6.13 | 0.001 | 0.034 | *Limosilactobacillus vaginalis* |
| ASV1101 | 29.3 | -5.47 | 0.002 | 0.043 | *Actinomyces* sp. |
| ASV1172 | 24.7 | -5.22 | 0.002 | 0.043 | *Prevotella timonensis* |
| ASV1199 | 22.5 | -5.09 | 0.003 | 0.043 | *Prevotella timonensis* |
| ASV1293 | 21.1 | -4.99 | 0.003 | 0.043 | *Ligilactobacillus animalis* |
| ASV1317 | 17.2 | -4.69 | 0.004 | 0.045 | *Streptococcus oralis* |
| ASV1347 | 16.6 | 4.66 | 0.004 | 0.045 | *Bifidobacterium longum* |
| ASV1378 | 15.5 | -4.54 | 0.005 | 0.049 | *Prevotella disiens* |
| ASV1390 | 18.2 | -4.78 | 0.004 | 0.045 | *Ligilactobacillus animalis* |
| ASV1466 | 13.9 | -4.37 | 0.007 | 0.049 | *Anaerococcus hydrogenalis* |
| ASV1519 | 13.9 | -4.38 | 0.006 | 0.049 | *Peptoniphilus indolicus* |
| ASV1586 | 17.1 | -4.68 | 0.004 | 0.045 | *Ligilactobacillus animalis* |
| ASV1692 | 14.5 | -4.44 | 0.006 | 0.049 | *Prevotella bivia* |

**Table S4.** **Differential relative abundance analysis (using DESeq2) of composition for 48 hours at room temperature compared with on receipt of sample.**

| **ASV** | **Base Mean** | **log2 Fold Change** | **p-value** | **adjusted p-value** | **Species** |
| --- | --- | --- | --- | --- | --- |
| ASV4 | 7592.0 | 9.09 | <0.001 | <0.001 | *Enterococcus faecalis* |
| ASV11 | 3775.1 | 12.58 | <0.001 | <0.001 | *Streptococcus agalactiae* |
| ASV19 | 261.7 | 8.74 | <0.001 | <0.001 | *Staphylococcus condimenti* |
| ASV29 | 573.6 | 9.87 | <0.001 | <0.001 | *Escherichia coli* |
| ASV33 | 424.8 | 6.10 | 0.001 | 0.009 | *Staphylococcus hominis* |
| ASV35 | 151.3 | 7.95 | <0.001 | 0.001 | *Enterococcus faecalis* |
| ASV42 | 7.4 | 3.53 | 0.011 | 0.047 | *Escherichia coli* |
| ASV50 | 11.4 | 4.19 | 0.005 | 0.030 | *Escherichia coli* |
| ASV69 | 573.0 | 6.42 | <0.001 | 0.003 | *Staphylococcus epidermidis* |
| ASV75 | 443.0 | 9.53 | <0.001 | <0.001 | *Staphylococcus haemolyticus* |
| ASV80 | 105.7 | -3.72 | 0.009 | 0.041 | *Peptoniphilus* sp. |
| ASV81 | 75.9 | 6.96 | <0.001 | 0.002 | *Staphylococcus carnosus* |
| ASV88 | 27.7 | 5.50 | 0.001 | 0.009 | *Escherichia coli* |
| ASV91 | 69.1 | 6.82 | <0.001 | 0.002 | *Staphylococcus condimenti* |
| ASV106 | 11.7 | -3.87 | 0.009 | 0.041 | *Peptoniphilus lacrimalis* |
| ASV123 | 82.6 | -6.77 | <0.001 | <0.001 | *Peptoniphilus indolicus* |
| ASV132 | 203.4 | 8.40 | <0.001 | <0.001 | *Staphylococcus haemolyticus* |
| ASV134 | 13.8 | -4.12 | 0.009 | 0.041 | *Peptoniphilus indolicus* |
| ASV139 | 131.1 | -4.71 | 0.006 | 0.032 | *Propionimicrobium lymphophilum* |
| ASV140 | 57.0 | 6.55 | <0.001 | 0.002 | *Staphylococcus hominis* |
| ASV155 | 145.9 | 7.90 | <0.001 | 0.000 | *Staphylococcus hominis* |
| ASV190 | 1306.2 | 11.05 | <0.001 | <0.001 | *Escherichia-Shigella* sp. |
| ASV195 | 1217.4 | 10.95 | <0.001 | <0.001 | *Shigella flexneri* |
| ASV209 | 85.9 | -6.83 | <0.001 | 0.003 | *Limosilactobacillus vaginalis* |
| ASV215 | 18.2 | 4.88 | 0.002 | 0.017 | *Escherichia coli* |
| ASV221 | 11.6 | -3.85 | 0.012 | 0.049 | *Schaalia turicensis* |
| ASV226 | 831.8 | 10.40 | <0.001 | <0.001 | *Enterococcus faecalis* |
| ASV233 | 23.3 | -4.91 | 0.003 | 0.022 | *Peptoniphilus indolicus* |
| ASV255 | 49.3 | -4.45 | 0.007 | 0.035 | *Anaerococcus obesiensis* |
| ASV267 | 167.3 | 8.10 | <0.001 | <0.001 | *Staphylococcus epidermidis* |
| ASV288 | 22.2 | 5.17 | 0.002 | 0.013 | *Escherichia coli* |
| ASV293 | 37.4 | -5.61 | 0.001 | 0.011 | *Anaerococcus hydrogenalis* |
| ASV304 | 568.9 | 9.85 | <0.001 | <0.001 | *Enterococcus faecalis* |
| ASV312 | 36.2 | -5.56 | 0.001 | 0.009 | *Varibaculum sp.* |
| ASV343 | 50.9 | -4.87 | 0.006 | 0.030 | *Veillonella parvula* |
| ASV349 | 34.3 | -5.49 | 0.002 | 0.013 | *Anaerococcus obesiensis* |
| ASV356 | 17.0 | 4.79 | 0.003 | 0.018 | *Staphylococcus haemolyticus* |
| ASV358 | 14.2 | -4.17 | 0.008 | 0.039 | *Dialister propionicifaciens* |
| ASV380 | 256.6 | 8.71 | <0.001 | <0.001 | *Streptococcus agalactiae* |
| ASV386 | 11.1 | -3.79 | 0.010 | 0.042 | *Schaalia* sp. |
| ASV390 | 111.2 | -7.20 | <0.001 | 0.002 | *Finegoldia magna* |
| ASV401 | 32.2 | -4.32 | 0.010 | 0.044 | *Actinotignum schaalii* |
| ASV417 | 179.4 | -5.13 | 0.010 | 0.042 | *Finegoldia magna* |
| ASV457 | 225.7 | 8.53 | <0.001 | <0.001 | *Staphylococcus epidermidis* |
| ASV488 | 21.1 | -4.76 | 0.004 | 0.024 | *Veillonella parvula* |
| ASV503 | 10.1 | 4.02 | 0.006 | 0.032 | *Staphylococcus simulans* |
| ASV511 | 158.0 | 8.01 | <0.001 | 0.001 | *Staphylococcus cohnii* |
| ASV512 | 147.2 | 7.91 | <0.001 | 0.001 | *Streptococcus agalactiae* |
| ASV513 | 203.5 | 8.38 | <0.001 | <0.001 | *Staphylococcus epidermidis* |
| ASV528 | 13.8 | 4.47 | 0.004 | 0.023 | *Staphylococcus epidermidis* |
| ASV541 | 123.2 | -7.35 | <0.001 | 0.002 | *Anaerococcus lactolyticus* |
| ASV548 | 20.2 | 5.04 | 0.002 | 0.014 | *Staphylococcus haemolyticus* |
| ASV587 | 19.0 | -3.87 | 0.012 | 0.049 | *Prevotella disiens* |
| ASV648 | 135.3 | 7.79 | <0.001 | 0.001 | *Staphylococcus epidermidis* |
| ASV739 | 26.4 | -5.10 | 0.003 | 0.018 | *Peptoniphilus coxii* |
| ASV745 | 66.5 | -6.45 | <0.001 | 0.004 | *Limosilactobacillus vaginalis* |
| ASV777 | 51.1 | -6.07 | 0.001 | 0.007 | *Bifidobacterium longum* |
| ASV793 | 63.7 | -5.20 | 0.004 | 0.023 | *Ligilactobacillus animalis* |
| ASV807 | 14.4 | 4.54 | 0.004 | 0.023 | *Escherichia coli* |
| ASV853 | 7.0 | 3.49 | 0.011 | 0.047 | *Staphylococcus haemolyticus* |
| ASV884 | 28.8 | 5.57 | 0.000 | 0.005 | *Staphylococcus epidermidis* |
| ASV931 | 44.8 | -5.88 | 0.001 | 0.009 | *Limosilactobacillus vaginalis* |
| ASV933 | 66.8 | 6.77 | <0.001 | 0.002 | *Staphylococcus hominis* |
| ASV987 | 59.5 | 6.60 | <0.001 | 0.003 | *Escherichia-Shigella sp.* |
| ASV1009 | 56.2 | 6.52 | <0.001 | 0.003 | *Staphylococcus hominis* |
| ASV1090 | 29.0 | -5.24 | 0.002 | 0.017 | *Bergeyella* sp. |
| ASV1101 | 28.6 | -5.22 | 0.002 | 0.017 | *Actinomyces* sp. |
| ASV1141 | 37.1 | 5.93 | <0.001 | 0.003 | *Staphylococcus haemolyticus* |
| ASV1172 | 24.1 | -4.96 | 0.003 | 0.021 | *Prevotella timonensis* |
| ASV1199 | 22.0 | -4.83 | 0.004 | 0.023 | *Prevotella timonensis* |
| ASV1293 | 20.7 | -4.73 | 0.004 | 0.024 | *Ligilactobacillus animalis* |
| ASV1317 | 16.9 | -4.43 | 0.006 | 0.032 | *Streptococcus oralis* |
| ASV1378 | 15.2 | -4.27 | 0.007 | 0.036 | *Prevotella disiens* |
| ASV1390 | 17.9 | -4.51 | 0.005 | 0.030 | *Ligilactobacillus animalis* |
| ASV1403 | 12.7 | -3.99 | 0.010 | 0.043 | *Prevotella disiens* |
| ASV1466 | 13.4 | -4.07 | 0.009 | 0.042 | *Anaerococcus hydrogenalis* |
| ASV1519 | 13.7 | -4.11 | 0.009 | 0.041 | *Peptoniphilus indolicus* |
| ASV1586 | 16.7 | -4.42 | 0.006 | 0.032 | *Ligilactobacillus animalis* |
| ASV1605 | 13.8 | 4.49 | 0.004 | 0.023 | *Staphylococcus haemolyticus* |
| ASV1692 | 14.2 | -4.17 | 0.008 | 0.039 | *Prevotella bivia* |
| ASV1824 | 13.0 | 4.38 | 0.004 | 0.025 | *Escherichia coli* |


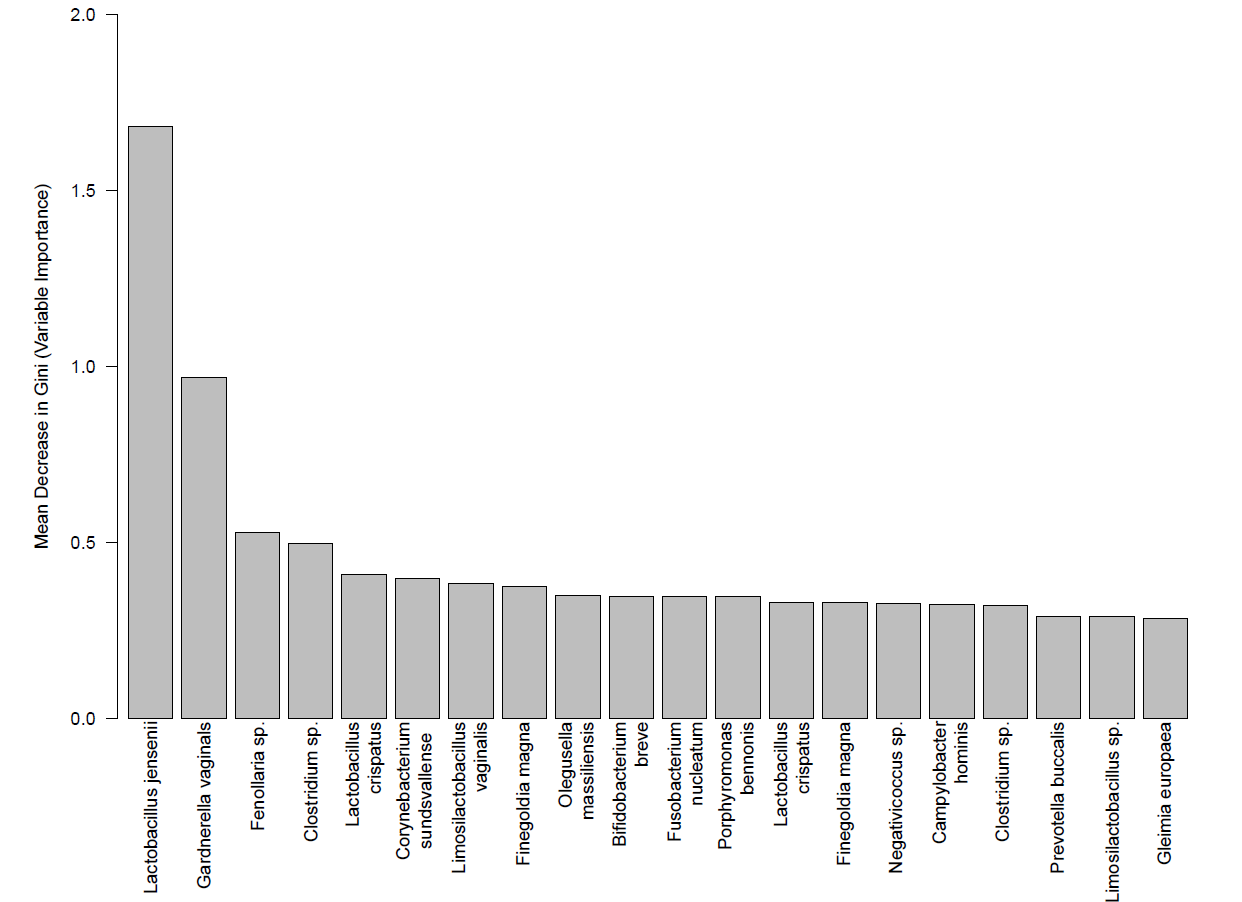


**Figure S10. ASV importance (mean decrease in Gini) for the 20 most important ASVs in the random forest model for differences between microbiota in vaginal swabs vs. vaginal-rectal swabs.** A greater decrease in Gini is indicative of a more important ASV in splitting the data by site sampled.

**Full R analysis code (R markdown script):**

```{r setup}

knitr::opts_chunk$set(

fig.cap.pre = "Figure ",

fig.cap.sep = ": ",

fig.cap.style = "Image Caption",

cache = TRUE,

tab.cap.pre = "Table ",

tab.cap.sep = ": ",

tab.cap.style = "Table Caption"

)

library(officedown)

library(officer)

library(ggplot2)

library(dplyr)

library(RColorBrewer)

library(gridExtra)

library(data.table)

library(stringr)

library(knitr)

library(tidyverse)

library(BiocManager)

#BiocManager::install("dada2")

library(dada2)

library(phyloseq)

library(dada2)

library(vegan)

library(doParallel)

library(foreach)

#Create a vector with today's date

timestamp <- Sys.time()

timestamp_formatted_full <- format(timestamp, format="%d-%b-%Y_%H%M")

timestamp_formatted <- format(timestamp, format="%d-%b-%Y")

operator_username <- system("whoami", intern=TRUE)

operator<- str_to_title(gsub('\\.', ' ', operator_username))

#create a folder for outputs with today's date (if not already present)

outputs_dir <- paste(getwd(),"/Outputs_",timestamp_formatted,sep="")

dir.create(outputs_dir, showWarnings = FALSE)

outputs_dir_slash <- paste(outputs_dir,"/", sep="")

current_dir <- getwd()

```

```{r dada2}

path <- "/group/tran4/Staff and students/Postdocs/Laura/Research Officer/GenV-GBS pilot project/Combined sequencing files/"

list.files(path)

# Forward and reverse fastq filenames have format: SAMPLENAME_R1_001.fastq and SAMPLENAME_R2_001.fastq

fnFs <- sort(list.files(path, pattern="_R1.fastq.gz", full.names = TRUE))

fnRs <- sort(list.files(path, pattern="_R2.fastq.gz", full.names = TRUE))

# Extract sample names, assuming filenames have format: SAMPLENAME_XXX.fastq.gz

sample.names <- sapply(strsplit(basename(fnFs), "_"), `[`, 1)

#### Inspect Quality of reads ####

plotQualityProfile(fnFs[1:3])

plotQualityProfile(fnRs[1:3])

#### Filter and trim ####

# Place filtered files in filtered/ subdirectory

filtFs <- file.path(path, "filtered", paste0(sample.names, "_F_filt.fastq.gz"))

filtRs <- file.path(path, "filtered", paste0(sample.names, "_R_filt.fastq.gz"))

names(filtFs) <- sample.names

names(filtRs) <- sample.names

out <- filterAndTrim(fnFs, filtFs, fnRs, filtRs,

maxN=0, maxEE=c(2,4), truncQ=2, trimLeft=c(20,18), truncLen =c(240,240), rm.phix=TRUE,

compress=TRUE, multithread=FALSE) # On Windows set multithread=FALSE

#Note: trimLeft used to remove primer sequences, both R1 and R2 had low quality in the primer region

head(out)

#visualise

plotQualityProfile(filtFs[1:3])

plotQualityProfile(filtRs[1:3])

#### Learn error rates ####

#Every amplicon dataset has own rate of errors

#learnErrors command learns the errors model from the data

#Alternates estimation of errors rates and inference of sample composition...

#...until they converge to a jointly consistent solution.

errF <- learnErrors(filtFs, multithread=TRUE)

errR <- learnErrors(filtRs, multithread=TRUE)

plotErrors(errF, nominalQ=TRUE)

plotErrors(errR, nominalQ=TRUE)

#### Sample inference ####

dadaFs <- dada(filtFs, err=errF, multithread=TRUE)

dadaRs <- dada(filtRs, err=errR, multithread=TRUE)

dadaFs[[1]]

dadaRs[[1]]

#### Merge paired reads ####

mergers <- mergePairs(dadaFs, filtFs, dadaRs, filtRs, verbose=TRUE)

# Inspect the merger data.frame from the first sample

head(mergers[[1]])

#### Construct Sequence table ####

seqtab <- makeSequenceTable(mergers)

dim(seqtab)

# Inspect distribution of sequence lengths

table(nchar(getSequences(seqtab)))

#remove sequences much shorter or longer than expected

seqtab2 <- seqtab[,nchar(colnames(seqtab)) %in% 305:345]

table(nchar(getSequences(seqtab2)))

#### Remove chimeras ####

seqtab.nochim <- removeBimeraDenovo(seqtab2, method="consensus", multithread=TRUE, verbose=TRUE)

seqtab.nochim2 <- removeBimeraDenovo(seqtab, method="consensus", multithread=TRUE, verbose=TRUE)

dim(seqtab.nochim)

dim(seqtab.nochim2)

#frequency of sequences remaining after removing chimeras

sum(seqtab.nochim)/sum(seqtab2)

#### Track reads through the pipeline ####

getN <- function(x) sum(getUniques(x))

track <- cbind(out, sapply(dadaFs, getN), sapply(dadaRs, getN), sapply(mergers, getN), rowSums(seqtab.nochim))

colnames(track) <- c("input", "filtered", "denoisedF", "denoisedR", "merged", "nonchim")

rownames(track) <- sample.names

head(track)

timestamp_formatted_full <- format(Sys.time(), format="%d-%b-%Y_%H%M")

filename <- paste0(outputs_dir_slash,"read_tracking_",timestamp_formatted_full,".csv")

write.csv(track, file = filename, row.names=TRUE)

#### Assign Taxonomy ####

taxa <- assignTaxonomy(seqtab.nochim, "/group/tran4/Bioinformatics_tools/DADA2_reference_files/silva_nr99_v138.1_wSpecies_train_set.fa.gz", multithread=TRUE)

taxa.print <- taxa # Removing sequence rownames for display only

rownames(taxa.print) <- NULL

head(taxa.print)

tax_ps1 <- phyloseq(otu_table(seqtab.nochim, taxa_are_rows=FALSE),

tax_table(taxa))

#rename to shorter strings

dna1 <- Biostrings::DNAStringSet(taxa_names(tax_ps1))

names(dna1) <- taxa_names(tax_ps1)

tax_ps1 <- merge_phyloseq(tax_ps1, dna1)

taxa_names(tax_ps1) <- paste0("ASV", seq(ntaxa(tax_ps1)))

tax_ps1

Taxon1_Table <- as.data.frame(tax_table(tax_ps1))

timestamp_formatted_full <- format(Sys.time(), format="%d-%b-%Y_%H%M")

filename <- paste0(outputs_dir_slash,"taxon_table_silva_",timestamp_formatted_full,".csv")

write.csv(Taxon1_Table, file = filename)

###Exploring RDP vs. Silva###

taxa2 <- assignTaxonomy(seqtab.nochim, "/group/tran4/Bioinformatics_tools/DADA2_reference_files/rdp_train_set_18.fa.gz", multithread=TRUE)

taxa2 <- addSpecies(taxa2, "/group/tran4/Bioinformatics_tools/DADA2_reference_files/rdp_species_assignment_18.fa.gz")

taxa2.print <- taxa2 # Removing sequence rownames for display only

rownames(taxa2.print) <- NULL

head(taxa2.print)

tax_ps2 <- phyloseq(otu_table(seqtab.nochim, taxa_are_rows=FALSE),

tax_table(taxa2))

#rename to shorter strings

dna2 <- Biostrings::DNAStringSet(taxa_names(tax_ps2))

names(dna2) <- taxa_names(tax_ps2)

tax_ps2 <- merge_phyloseq(tax_ps2, dna2)

taxa_names(tax_ps2) <- paste0("ASV", seq(ntaxa(tax_ps2)))

tax_ps2

Taxon2_Table <- as.data.frame(tax_table(tax_ps2))

timestamp_formatted_full <- format(Sys.time(), format="%d-%b-%Y_%H%M")

filename <- paste0(outputs_dir_slash,"taxon_table_rdp_",timestamp_formatted_full,".csv")

write.csv(Taxon2_Table, file = filename)

```

```{r data-organising-from-dada2}

##From DADA2 after taxonomy step:

ps1 <- phyloseq(otu_table(seqtab.nochim, taxa_are_rows=FALSE),

tax_table(taxa))

#rename to shorter strings

dna <- Biostrings::DNAStringSet(taxa_names(ps1))

names(dna) <- taxa_names(ps1)

ps1 <- merge_phyloseq(ps1, dna)

taxa_names(ps1) <- paste0("ASV", seq(ntaxa(ps1)))

ps1

##Get tables

Count_Table <- as.data.frame(otu_table(ps1))

timestamp_formatted_full <- format(Sys.time(), format="%d-%b-%Y_%H%M")

filename <- paste0(outputs_dir_slash,"count_table_",timestamp_formatted_full,".csv")

write.csv(Count_Table, file = filename)

Taxon_Table <- as.data.frame(tax_table(ps1))

timestamp_formatted_full <- format(Sys.time(), format="%d-%b-%Y_%H%M")

filename <- paste0(outputs_dir_slash,"taxon_table_",timestamp_formatted_full,".csv")

write.csv(Taxon_Table, file = filename)

RefSeq_Table <- as.data.frame(refseq(ps1))

timestamp_formatted_full <- format(Sys.time(), format="%d-%b-%Y_%H%M")

filename <- paste0(outputs_dir_slash,"refseq_table_",timestamp_formatted_full,".csv")

write.csv(RefSeq_Table, file = filename)

```

```{r phylogenetic-trees}

library(BiocManager)

#BiocManager::install("msa")

library(msa)

##Add a phylogenetic tree

seqs <- DNAStringSet(getSequences(refseq(ps1)), use.names=TRUE)

head(seqs)

mult <- msa(seqs, method="ClustalW", type="dna", order="input")

library("phangorn")

phang.align <- as.phyDat(mult, type="DNA", names=getSequence(refseq(ps1)))

dm <- dist.ml(phang.align)

treeNJ <- NJ(dm) # Note, tip order != sequence order

fit = pml(treeNJ, data=phang.align)

## negative edges length changed to 0!

fitGTR <- update(fit, k=4, inv=0.2)

fitGTR <- optim.pml(fitGTR, model="GTR", optInv=TRUE, optGamma=TRUE,

rearrangement = "stochastic", control = pml.control(trace = 0))

detach("package:phangorn", unload=TRUE)

phy_tree(ps1) <- phy_tree(fitGTR$tree)

ps1

```

```{r add-sample-data-and-cleaned-taxa}

#load sample information

sample_info <- read.csv("/group/tran4/Staff and students/Postdocs/Laura/Research Officer/GenV-GBS pilot project/Combined sequencing analysis/sample_data.csv", row.names=1)

sample_data(ps1) <- sample_data(sample_info)

View(sample_data(ps1))

##Clean-up output of taxa file and reload:

ps2 <- ps1

ps2 <- prune_samples(sample_sums(ps1) > 0, ps1)

ps2

taxid_edit <- read.csv("/group/tran4/Staff and students/Postdocs/Laura/Research Officer/GenV-GBS pilot project/Combined sequencing analysis/taxon_table_30Nov21_format.csv", row.names=1)

taxid_ed2 <- as.matrix(taxid_edit)

tax_table(ps2) <- tax_table(taxid_ed2)

```

```{r understand-potential-contaminants}

library(BiocManager)

#BiocManager::install("decontam")

library(decontam)

df <- as.data.frame(sample_data(ps2)) # Put sample_data into a ggplot-friendly data.frame

df$LibrarySize <- sample_sums(ps2)

df <- df[order(df$LibrarySize),]

df$Index <- seq(nrow(df))

ggplot(data=df, aes(x=Index, y=LibrarySize, color=for_decontam)) + geom_point()

#contamination by frequency

contamdf.freq <- isContaminant(ps2, method="frequency", conc="Concentration")

head(contamdf.freq)

Contam_Freq <- as.data.frame(contamdf.freq)

timestamp_formatted_full <- format(Sys.time(), format="%d-%b-%Y_%H%M")

filename <- paste0(outputs_dir_slash,"contam_freq_",timestamp_formatted_full,".csv")

write.csv(Contam_Freq, file = filename)

table(contamdf.freq$contaminant)

head(which(contamdf.freq$contaminant))

plot_frequency(ps2, taxa_names(ps2)[c(1,606)], conc="Concentration") +

xlab("DNA Concentration ")

set.seed(100)

plot_frequency(ps2, taxa_names(ps2)[sample(which(contamdf.freq$contaminant),6)], conc="Concentration") +

xlab("DNA Concentration")

#contamination by prevalence

sample_data(ps2)$is.neg <- sample_data(ps2)$for_decontam == "Negative control"

contamdf.prev <- isContaminant(ps2, method="prevalence", neg="is.neg")

table(contamdf.prev$contaminant)

Contam_Prev <- as.data.frame(contamdf.prev)

timestamp_formatted_full <- format(Sys.time(), format="%d-%b-%Y_%H%M")

filename <- paste0(outputs_dir_slash,"contam_prev_",timestamp_formatted_full,".csv")

write.csv(Contam_Prev, file = filename)

head(which(contamdf.prev$contaminant))

contamdf.prev05 <- isContaminant(ps2, method="prevalence", neg="is.neg", threshold=0.5)

table(contamdf.prev05$contaminant)

# Make phyloseq object of presence-absence in negative controls and true samples

ps.pa <- transform_sample_counts(ps2, function(abund) 1*(abund>0))

ps.pa.neg <- prune_samples(sample_data(ps.pa)$for_decontam == "Negative control", ps.pa)

ps.pa.pos <- prune_samples(sample_data(ps.pa)$for_decontam == "True sample", ps.pa)

# Make data.frame of prevalence in positive and negative samples

df.pa <- data.frame(pa.pos=taxa_sums(ps.pa.pos), pa.neg=taxa_sums(ps.pa.neg),

contaminant=contamdf.prev$contaminant)

ggplot(data=df.pa, aes(x=pa.neg, y=pa.pos, color=contaminant)) + geom_point() +

xlab("Prevalence (Negative Controls)") + ylab("Prevalence (True Samples)")

#combined results

contamdf.comb <- isContaminant(ps2, method="combined", conc="Concentration", neg="is.neg")

table(contamdf.comb$contaminant)

head(which(contamdf.comb$contaminant))

Contam_Comb <- as.data.frame(contamdf.comb)

timestamp_formatted_full <- format(Sys.time(), format="%d-%b-%Y_%H%M")

filename <- paste0(outputs_dir_slash,"contam_comb_",timestamp_formatted_full,".csv")

write.csv(Contam_Comb, file = filename)

plot_frequency(ps2, taxa_names(ps2)[c(1,19,809,1894)], conc="Concentration") +

xlab("DNA Concentration ")

```

```{r data-prep-for-analysis }

ps2.prop <- transform_sample_counts(ps2, function(otu) otu/sum(otu))

ps2.prop100 <- transform_sample_counts(ps2, function(otu) otu/sum(otu)*100)

#### Subsetting samples ####

## Stage One only ##

S1 <- subset_samples(ps2, Stage == "One")

S1 <- prune_taxa(taxa_sums(S1) > 0, S1)

S1.prop <- transform_sample_counts(S1, function(otu) otu/sum(otu))

S1.prop100 <- transform_sample_counts(S1, function(otu) otu/sum(otu)*100)

S1

#samples only

S1_samples <- subset_samples(S1, Sample.Type == "Sample")

S1_samples <- prune_taxa(taxa_sums(S1_samples) > 0, S1_samples)

S1_samples.prop <- transform_sample_counts(S1_samples, function(otu) otu/sum(otu))

S1_samples.prop100 <- transform_sample_counts(S1_samples, function(otu) otu/sum(otu)*100)

S1_samples

#controls only

S1_controls <- subset_samples(S1, Sample.Type == "Control")

S1_controls <- prune_taxa(taxa_sums(S1_controls) > 0, S1_controls)

S1_controls.prop <- transform_sample_counts(S1_controls, function(otu) otu/sum(otu))

S1_controls.prop100 <- transform_sample_counts(S1_controls, function(otu) otu/sum(otu)*100)

S1_controls

plot_bar(S1_controls)

#replicates only - two different DNA extraction methods

S1_reps <- subset_samples(S1, SampleReplicate == "Yes")

S1_reps <- prune_taxa(taxa_sums(S1_reps) > 0, S1_reps)

S1_reps.prop <- transform_sample_counts(S1_reps, function(otu) otu/sum(otu))

S1_reps.prop100 <- transform_sample_counts(S1_reps, function(otu) otu/sum(otu)*100)

S1_reps

#No replicates - DNA extraction method 4 only

S1_m4 <- subset_samples(S1_samples, ExtractionMethod == "M4")

S1_m4 <- prune_taxa(taxa_sums(S1_m4) > 0, S1_m4)

S1_m4.prop <- transform_sample_counts(S1_m4, function(otu) otu/sum(otu))

S1_m4.prop100 <- transform_sample_counts(S1_m4, function(otu) otu/sum(otu)*100)

S1_m4

## Stage Two only ##

S2 <- subset_samples(ps2, Stage == "Two")

S2 <- prune_taxa(taxa_sums(S2) > 0, S2)

#SH15D01 failed QC - remove here from analyses

S2 <- subset_samples(S2, row.names(sample_data(S2))!="SH15D01")

S2 <- prune_taxa(taxa_sums(S2) > 0, S2)

S2.prop <- transform_sample_counts(S2, function(otu) otu/sum(otu))

S2.prop100 <- transform_sample_counts(S2, function(otu) otu/sum(otu)*100)

S2

#samples only

S2_samples <- subset_samples(S2, Sample.Type == "Sample")

S2_samples <- prune_taxa(taxa_sums(S2_samples) > 0, S2_samples)

S2.samples.prop <- transform_sample_counts(S2_samples, function(otu) otu/sum(otu))

S2.samples.prop100 <- transform_sample_counts(S2_samples, function(otu) otu/sum(otu)*100)

S2_samples

#controls only

S2_controls <- subset_samples(S2, Sample.Type == "Control")

S2_controls <- prune_taxa(taxa_sums(S2_controls) > 0, S2_controls)

S2.controls.prop <- transform_sample_counts(S2_controls, function(otu) otu/sum(otu))

S2.controls.prop100 <- transform_sample_counts(S2_controls, function(otu) otu/sum(otu)*100)

S2_controls

plot_bar(S2_controls)

# 4 degrees only

S2_4d <- subset_samples(S2_samples, Condition == "4 degrees")

S2_4d <- prune_taxa(taxa_sums(S2_4d) > 0, S2_4d)

S2.4d.prop <- transform_sample_counts(S2_4d, function(otu) otu/sum(otu))

S2.4d.prop100 <- transform_sample_counts(S2_4d, function(otu) otu/sum(otu)*100)

S2_4d

#room temperature only

S2_rt <- subset_samples(S2_samples, Condition == "room temp")

S2_rt <- prune_taxa(taxa_sums(S2_rt) > 0, S2_rt)

S2.rt.prop <- transform_sample_counts(S2_rt, function(otu) otu/sum(otu))

S2.rt.prop100 <- transform_sample_counts(S2_rt, function(otu) otu/sum(otu)*100)

S2_rt

##Positive controls -check

Count_PCs <- subset_samples(ps2, Sample.Description == "PositiveControl")

PCs <- prune_taxa(taxa_sums(PCs) > 0, PCs)

PCs.prop <- transform_sample_counts(PCs, function(otu) otu/sum(otu))

PCs.prop100 <- transform_sample_counts(PCs, function(otu) otu/sum(otu)*100)

PCs

plot_bar(PCs.prop100, fill="Species")

Count_Table_PCs <- as.data.frame(otu_table(PCs))

timestamp_formatted_full <- format(Sys.time(), format="%d-%b-%Y_%H%M")

filename <- paste0(outputs_dir_slash,"count_table_PCs_",timestamp_formatted_full,".csv")

write.csv(Count_Table_PCs, file = filename)

Taxon_Table_PCs <- as.data.frame(tax_table(PCs))

timestamp_formatted_full <- format(Sys.time(), format="%d-%b-%Y_%H%M")

filename <- paste0(outputs_dir_slash,"taxon_table_PCs_",timestamp_formatted_full,".csv")

write.csv(Taxon_Table_PCs, file = filename)

Abund_Table_PCs <- as.data.frame(otu_table(PCs.prop100))

timestamp_formatted_full <- format(Sys.time(), format="%d-%b-%Y_%H%M")

filename <- paste0(outputs_dir_slash,"abund_table_PCs_",timestamp_formatted_full,".csv")

write.csv(Abund_Table_PCs, file = filename)

### Others ###

#plot most abundant taxa

taxatab <- as.data.frame(otu_table(ps2_samples))

maxtaxab <- apply(taxatab, 2, max)

toptaxab <- apply(taxatab, 2, sum)

head(maxtaxab)

head(toptaxab)

barplot(sort(maxtaxab,TRUE)[1:30],las=2, col="blue", border=NA, yaxp=c(0,125000,5), main="Top 20 ranked by max abundance")

barplot(sort(toptaxab,TRUE)[1:30],las=2, col="blue", border=NA, yaxp=c(0,2000000,4), main="Top 20 ranked by total abundance")

barplot(sort(toptaxab,TRUE)[1:30],las=2, col="blue", border=NA, log="y", ylim=c(5e4,1e7), main="Top 20 ranked by total abundance")

````

```{stage-one}

sample_names(S1_m4) <- c("GBS06","GBS07","GBS08","GBS09", "GBS10","GBS11","GBS12","GBS13","GBS14","GBS15","GBS16","GBS17","GBS18","GBS19", "GBS20","GBS21","GBS22","GBS23","GBS24","GBS25")

sample_names(S1_m4.prop) <- c("GBS06","GBS07","GBS08","GBS09", "GBS10","GBS11","GBS12","GBS13","GBS14","GBS15","GBS16","GBS17","GBS18","GBS19", "GBS20","GBS21","GBS22","GBS23","GBS24","GBS25")

sample_names(S1_m4.prop100) <- c("GBS06","GBS07","GBS08","GBS09", "GBS10","GBS11","GBS12","GBS13","GBS14","GBS15","GBS16","GBS17","GBS18","GBS19", "GBS20","GBS21","GBS22","GBS23","GBS24","GBS25")

#### Alpha-diversity ####

s1_rich_p <- plot_richness(S1_m4, measures = c("Observed", "shannon", "simpson")) +

labs(x="") + theme_bw() +

theme(axis.text.x=element_text(angle=270, hjust=1, vjust=0.5, size=7))

timestamp_formatted_full <- format(Sys.time(), format="%d-%b-%Y_%H%M")

filename <- paste0("p_S1_richness_",timestamp_formatted_full,".png")

ggsave(filename, plot=s1_rich_p, device = png,

path = outputs_dir,

width=16.7, height=8, units="cm", dpi = 600, limitsize = TRUE)

s1_rich <- estimate_richness(S1_m4)

timestamp_formatted_full <- format(Sys.time(), format="%d-%b-%Y_%H%M")

filename <- paste0(outputs_dir_slash,"S1_richness_",timestamp_formatted_full,".csv")

write.csv(s1_rich, file = filename)

#### Beta-diversity ####

#unweighted UniFrac

#for presence/absence of taxa between samples

ps.samUF1m4 <- UniFrac(S1_m4.prop)

ps.pcoa1m4 = ordinate(S1_m4.prop, method="PCoA", distance=ps.samUF1m4)

ps.pcoa1m4

#Visualise importance of each axis

plot_scree(ps.pcoa1m4, "Scree plot, UniFrac/PCoA")

(samuw1m4 <- plot_ordination(S1_m4.prop, ps.pcoa1m4, "samples", color="SampleLabel", title ="unweightedUniFrac") +

geom_point(size=3) )

(samuw1m4 <- plot_ordination(S1_m4.prop, ps.pcoa1m4, "samples", color="Lactobacillus_sp", title ="unweightedUniFrac") +

geom_point(size=3) )

#weighted UniFrac

#for differences of abundance in taxa between sample - takes longer

ps.sam.wUF1m4 <- UniFrac(S1_m4.prop, weighted = TRUE, normalized = TRUE)

ps.wpcoa1m4 = ordinate(S1_m4.prop, method="PCoA", distance=ps.sam.wUF1m4)

ps.wpcoa1m4

#Visualise importance of each axis

plot_scree(ps.wpcoa1m4, "Scree plot, wUniFrac/PCoA")

(samw1 <- plot_ordination(S1_m4.prop, ps.wpcoa1m4, "samples", color="SampleLabel", title="weighted UniFrac") +

geom_point(size=3))

(samw1 <- plot_ordination(S1_m4.prop, ps.wpcoa1m4, "samples", color="Lactobacillus_sp", title="weighted UniFrac") +

geom_point(size=3))

#Bray, NMDS - for abundance

ord.nmds.bray.s1m4 <- ordinate(S1_m4.prop, method="NMDS", distance="bray")

ord.nmds.bray.s1m4

stressplot(ord.nmds.bray.s1m4)

(sambr1m4 <-plot_ordination(S1_m4.prop, ord.nmds.bray.s1m4, "samples", title="Bray NMDS", color="SampleLabel") +

geom_point(size=3))

(sambr1Lm4 <-plot_ordination(S1_m4.prop, ord.nmds.bray.s1m4, "samples", title="Bray NMDS", color="Lactobacillus_sp") +

geom_point(size=3))

#Jaccard, NMDS - presence/absence

ord.nmds.jac.s1m4 <- ordinate(S1_m4.prop, method="NMDS", distance="jaccard", binary=TRUE)

ord.nmds.jac.s1m4

stressplot(ord.nmds.jac.s1m4)

(samja1m4 <- plot_ordination(S1_m4.prop, ord.nmds.jac.s1m4, title="Jaccard NMDS", type = "samples", color="SampleLabel") +

geom_point(size=3))

(samja1Lm4 <-plot_ordination(S1_m4.prop, ord.nmds.jac.s1m4, "samples", title="Bray NMDS", color="Lactobacillus_sp") +

geom_point(size=3))

#### Bar graphs ####

#Only samples

top100sam.s1m4 <- names(sort(taxa_sums(S1_m4), decreasing=TRUE))[1:100]

ps.top100sam.s1m4 <- prune_taxa(top100sam.s1m4, S1_m4.prop100)

top100genus <- plot_bar(ps.top100sam.s1m4, fill="Genus", x="SampleLabel") +

theme_bw() +

theme(axis.text.x = element_text(angle=270,hjust=0, vjust=0.5),

legend.text = element_text(size=10, face="italic"),

legend.key.size = unit(0.3, "cm")) +

labs(y = "Relative Abundance (%)", x ="") +

geom_bar(stat="identity") +

guides(fill = guide_legend(ncol = 2))

timestamp_formatted_full <- format(Sys.time(), format="%d-%b-%Y_%H%M")

filename <- paste0("p_S1_top100genus_",timestamp_formatted_full,".png")

ggsave(filename, plot=top100genus, device = png,

path = outputs_dir,

width=15.88, height=10.7, units="cm", dpi = 600, limitsize = TRUE)

top50sam.s1m4 <- names(sort(taxa_sums(S1_m4), decreasing=TRUE))[1:50]

ps.top50sam.s1m4 <- prune_taxa(top50sam.s1m4, S1_m4.prop100)

top50genus <- plot_bar(ps.top50sam.s1m4, fill="Genus", x="SampleLabel") +

theme_bw() +

theme(axis.text.x = element_text(angle=270,hjust=0, vjust=0.5),

legend.text = element_text(size=10,face="italic"),

legend.key.size = unit(0.4, "cm")) +

labs(y = "Relative Abundance (%)", x ="") +

geom_bar(stat="identity") +

guides(fill = guide_legend(ncol = 1))

timestamp_formatted_full <- format(Sys.time(), format="%d-%b-%Y_%H%M")

filename <- paste0("p_S1_top50genus_",timestamp_formatted_full,".png")

ggsave(filename, plot=top50genus, device = png,

path = outputs_dir,

width=15.88, height=10.7, units="cm", dpi = 600, limitsize = TRUE)

#Lactobacillus

S1m4_sam_lacto <- subset_taxa(S1_m4.prop100, Genus == "Lactobacillus")

my_legend_title <- expression(paste(italic("Lactobacillus"), " species", sep=""))

pl <- plot_bar(S1m4_sam_lacto, fill="Species", x="SampleLabel") +

theme_bw() +

theme(axis.text.x = element_text(angle=270, hjust=0, vjust=0.5),

legend.text = element_text(size=10, face="italic")) +

labs(y = "Relative Abundance (%)", x ="") +

geom_bar(stat="identity") +

scale_fill_discrete(name = my_legend_title, labels = c("crispatus", "gasseri", "helveticus","iners","jensenii","paragasseri","rogosae","taiwanensis"))

timestamp_formatted_full <- format(Sys.time(), format="%d-%b-%Y_%H%M")

filename <- paste0("p_S1_lactobacillus_",timestamp_formatted_full,".png")

ggsave(filename, plot=pl, device = png,

path = outputs_dir,

width=15.88, height=7.5, units="cm", dpi = 600, limitsize = TRUE)

```

```{stage-two}

#### Alpha diversity ####

## 4 degrees ##

# x-axis title - with degrees symbol

xlab = expression(Days~at~"4"*degree*C)

# Box plot of alpha-diversity measures

S2_4d_rich_box <- plot_richness(S2_4d, x="Timing", measures = c("Observed","shannon", "simpson"), color = "Timing")+ geom_boxplot(fill=NA) + theme_bw() + theme(axis.text.x = element_text(angle=270,hjust=0, vjust=0.5)) +labs(x=xlab, color="Sample") + scale_x_discrete(labels=c("0 days" = "0 days", "01 day" = "1 day", "05 days" = "5 days", "10 days"="10 days"))

timestamp_formatted_full <- format(Sys.time(), format="%d-%b-%Y_%H%M")

filename <- paste0("p_S2_rich_4d_box_",timestamp_formatted_full,".png")

ggsave(filename, plot=S2_4d_rich_box, device = png,

path = outputs_dir,

width=15.88, height=11, units="cm", dpi = 600, limitsize = TRUE)

# Line plot for each sample of alpha-diversity measures

S2_4d_rich_line <- plot_richness(S2_4d, x="Timing", measures = c("Observed","shannon", "simpson"), color = "SampleLabel")+ geom_path(aes(group=SampleLabel)) + theme_bw() + theme(axis.text.x = element_text(angle=270,hjust=0, vjust=0.5)) +labs(x=xlab, color="Sample") + scale_x_discrete(labels=c("0 days" = "0 days", "01 day" = "1 day", "05 days" = "5 days", "10 days"="10 days"))

timestamp_formatted_full <- format(Sys.time(), format="%d-%b-%Y_%H%M")

filename <- paste0("p_S2_rich_4d_line_",timestamp_formatted_full,".png")

ggsave(filename, plot=S2_4d_rich_line, device = png,

path = outputs_dir,

width=15.88, height=11, units="cm", dpi = 600, limitsize = TRUE)

# Save the measures

rich_4d <- estimate_richness(S2_4d)

timestamp_formatted_full <- format(Sys.time(), format="%d-%b-%Y_%H%M")

filename <- paste0(outputs_dir_slash,"S2_richness4d_",timestamp_formatted_full,".csv")

write.csv(rich_4d, file = filename)

## Room temperature ##

S2_rt_rich_box <- plot_richness(S2_rt, x="Timing", measures = c("Observed","shannon", "simpson"), color = "Timing") + geom_boxplot(fill=NA) + theme_bw() + theme(axis.text.x = element_text(angle=270,hjust=0, vjust=0.5)) + scale_fill_discrete(name ="Sample")

timestamp_formatted_full <- format(Sys.time(), format="%d-%b-%Y_%H%M")

filename <- paste0("p_S2_rich_rt_box_",timestamp_formatted_full,".png")

ggsave(filename, plot=S2_rt_rich_box, device = png,

path = outputs_dir,

width=15.88, height=11, units="cm", dpi = 600, limitsize = TRUE)

S2_rt_rich_line <- plot_richness(S2_rt, x="Timing", measures = c("Observed","shannon", "simpson"), color = "SampleLabel") + geom_line(aes(group=SampleLabel))+ theme_bw() + theme(axis.text.x = element_text(angle=270,hjust=0, vjust=0.5)) + labs(x="Hours at room temperature", color="Sample") + scale_x_discrete(labels=c("0 hours" = "0 hours", "04 hours" = "4 hours", "24 hours" = "24 hours", "48 hours"="48 hours"))

timestamp_formatted_full <- format(Sys.time(), format="%d-%b-%Y_%H%M")

filename <- paste0("p_S2_rich_rt_line_",timestamp_formatted_full,".png")

ggsave(filename, plot=S2_rt_rich_line, device = png,

path = outputs_dir,

width=15.88, height=11, units="cm", dpi = 600, limitsize = TRUE)

rich_rt <- estimate_richness(S2_rt)

timestamp_formatted_full <- format(Sys.time(), format="%d-%b-%Y_%H%M")

filename <- paste0(outputs_dir_slash,"S2_richnessrt_",timestamp_formatted_full,".csv")

write.csv(rich_rt, file = filename)

plot_richness(S2_samples, x="Location.of.sample", measures = c("Observed","shannon", "simpson"), color = "Location.of.sample") + geom_boxplot(fill=NA)

plot_richness(S2_samples, x="Extraction.batch", measures = c("Observed","shannon", "simpson"), color = "Extraction.batch") + geom_boxplot(fill=NA)

plot_richness(S2_samples, x="Method.of.collection", measures = c("Observed","shannon", "simpson"), color = "Method.of.collection") + geom_boxplot(fill=NA)

rich_S2 <- estimate_richness(S2_samples)

timestamp_formatted_full <- format(Sys.time(), format="%d-%b-%Y_%H%M")

filename <- paste0(outputs_dir_slash,"S2_richness_",timestamp_formatted_full,".csv")

write.csv(rich_S2, file = filename)

#### Beta-diversity ####

### All samples combined ###

#unweighted UniFrac

#for presence/absence of taxa between samples

S2.UF.sam <- UniFrac(S2_samples)

S2.pcoa.sam = ordinate(S2_samples, method="PCoA", distance=S2.UF.sam)

S2.pcoa.sam

#Visualise importance of each axis

plot_scree(S2.pcoa.sam, "Scree plot, UniFrac/PCoA")

S2uwUFPC = plot_ordination(S2.samples.prop, S2.pcoa.sam, color="SampleLabel", shape = "Condition", title ="unweighted UniFrac") +

geom_point(size=3) + stat_ellipse(type="norm", alpha=0.4)+ labs(color="Sample") +theme_bw()

timestamp_formatted_full <- format(Sys.time(), format="%d-%b-%Y_%H%M")

filename <- paste0("p_S2_unweighted_UniFrac_PCoA_",timestamp_formatted_full,".png")

ggsave(filename, plot=S2uwUFPC, device = png,

path = outputs_dir,

width=19.5, height=15.6, units="cm", dpi = 600, limitsize = TRUE)

#weighted UniFrac

#for differences of abundance in taxa between sample - takes longer

S2.wUF.sam <- UniFrac(S2_samples, weighted = TRUE, normalized = TRUE)

S2.wpcoa.sam = ordinate(S2_samples, method="PCoA", distance=S2.wUF.sam)

S2.wpcoa.sam

#Visualise importance of each axis

plot_scree(S2.wpcoa.sam, "Scree plot, wUniFrac/PCoA")

S2wUFPC = plot_ordination(S2.samples.prop, S2.wpcoa.sam, color="SampleLabel", shape = "Condition", title="weighted UniFrac") +

geom_point(size=3) + stat_ellipse(type="norm", alpha=0.4) + labs(color="Sample") +theme_bw()

timestamp_formatted_full <- format(Sys.time(), format="%d-%b-%Y_%H%M")

filename <- paste0("p_S2_weighted_UniFrac_PCoA_",timestamp_formatted_full,".png")

ggsave(filename, plot=S2wUFPC, device = png,

path = outputs_dir,

width=19.5, height=15.6, units="cm", dpi = 600, limitsize = TRUE)

#Bray, NMDS - for abundance

S2.ord.nmds.bray <- ordinate(S2.samples.prop, method="NMDS", distance="bray")

S2.ord.nmds.bray

stressplot(S2.ord.nmds.bray)

S2.bray <- plot_ordination(S2.samples.prop, S2.ord.nmds.bray, title="Bray-Curtis NMDS", color="SampleLabel", shape="Condition") +

geom_point(size=3) + stat_ellipse(type="norm", alpha=0.4) + labs(color="Sample") + theme_bw()

timestamp_formatted_full <- format(Sys.time(), format="%d-%b-%Y_%H%M")

filename <- paste0("p_S2_bray_nmds_",timestamp_formatted_full,".png")

ggsave(filename, plot=S2.bray, device = png,

path = outputs_dir,

width=19.5, height=15.6, units="cm", dpi = 600, limitsize = TRUE)

#Jaccard, NMDS - presence/absence

S2.ord.nmds.jac <- ordinate(S2.samples.prop, method="NMDS", distance="jaccard", binary=TRUE)

S2.ord.nmds.jac

stressplot(S2.ord.nmds.jac)

S2.jacc <- plot_ordination(S2.samples.prop, S2.ord.nmds.jac, title="Jaccard NMDS", color="SampleLabel", shape="Condition") + geom_point(size=3) + stat_ellipse(type="norm", alpha=0.4) + labs(color="Sample") + theme_bw()

timestamp_formatted_full <- format(Sys.time(), format="%d-%b-%Y_%H%M")

filename <- paste0("p_S2_jaccard_nmds_",timestamp_formatted_full,".png")

ggsave(filename, plot=S2.jacc, device = png,

path = outputs_dir,

width=19.5, height=15.6, units="cm", dpi = 600, limitsize = TRUE)

##Testing variables - 4d

bray_dist_4d = phyloseq::distance(S2_4d, method="bray")

jacc_dist_4d = phyloseq::distance(S2_4d, method="jaccard", binary=TRUE)

unwu_dist_4d = phyloseq::distance(S2_4d, method="unifrac")

wu_dist_4d = phyloseq::distance(S2_4d, method="wunifrac")

#Storage at 4 degrees

perm_4d_b <- adonis(bray_dist_4d ~ sample_data(S2_4d)$'Timing')

perm_4d_b

timestamp_formatted_full <- format(Sys.time(), format="%d-%b-%Y_%H%M")

filename <- paste0(outputs_dir_slash,"PERMANOVA_4d_conditions_bray_",timestamp_formatted_full,".csv")

write.csv(perm_4d_b$aov.tab, file = filename)

perm_4d_j <- adonis(jacc_dist_4d ~ sample_data(S2_4d)$'Timing')

perm_4d_j

timestamp_formatted_full <- format(Sys.time(), format="%d-%b-%Y_%H%M")

filename <- paste0(outputs_dir_slash,"PERMANOVA_4d_conditions_jaccard_",timestamp_formatted_full,".csv")

write.csv(perm_4d_j$aov.tab, file = filename)

perm_4d_uni <- adonis(unwu_dist_4d ~ sample_data(S2_4d)$'Timing')

perm_4d_uni

timestamp_formatted_full <- format(Sys.time(), format="%d-%b-%Y_%H%M")

filename <- paste0(outputs_dir_slash,"PERMANOVA_4d_conditions_unweightedUniFrac_",timestamp_formatted_full,".csv")

write.csv(perm_4d_uni$aov.tab, file = filename)

perm_4d_wuni <- adonis(wu_dist_4d ~ sample_data(S2_4d)$'Timing')

perm_4d_wuni

timestamp_formatted_full <- format(Sys.time(), format="%d-%b-%Y_%H%M")

filename <- paste0(outputs_dir_slash,"PERMANOVA_4d_conditions_weightedUniFrac_",timestamp_formatted_full,".csv")

write.csv(perm_4d_wuni$aov.tab, file = filename)

##Testing variables##

bray_dist_rt = phyloseq::distance(S2_rt, method="bray")

jacc_dist_rt = phyloseq::distance(S2_rt, method="jaccard", binary=TRUE)

unwu_dist_rt = phyloseq::distance(S2_rt, method="unifrac")

wu_dist_rt = phyloseq::distance(S2_rt, method="wunifrac")

#Storage at room temperature

perm_rt_b <- adonis(bray_dist_rt ~ sample_data(S2_rt)$'Timing')

perm_rt_b

mestamp_formatted_full <- format(Sys.time(), format="%d-%b-%Y_%H%M")

filename <- paste0(outputs_dir_slash,"PERMANOVA_rt_conditions_bray_",timestamp_formatted_full,".csv")

write.csv(perm_rt_b$aov.tab, file = filename)

perm_rt_j <- adonis(jacc_dist_rt ~ sample_data(S2_rt)$'Timing')

perm_rt_j

timestamp_formatted_full <- format(Sys.time(), format="%d-%b-%Y_%H%M")

filename <- paste0(outputs_dir_slash,"PERMANOVA_rt_conditions_jaccard_",timestamp_formatted_full,".csv")

write.csv(perm_rt_j$aov.tab, file = filename)

perm_rt_uni <- adonis(unwu_dist_rt ~ sample_data(S2_rt)$'Timing')

perm_rt_uni

filename <- paste0(outputs_dir_slash,"PERMANOVA_rt_conditions_unweightedUniFrac_",timestamp_formatted_full,".csv")

write.csv(perm_rt_uni$aov.tab, file = filename)

perm_rt_wuni <- adonis(wu_dist_rt ~ sample_data(S2_rt)$'Timing')

perm_rt_wuni

timestamp_formatted_full <- format(Sys.time(), format="%d-%b-%Y_%H%M")

filename <- paste0(outputs_dir_slash,"PERMANOVA_rt_conditions_weightedUniFrac_",timestamp_formatted_full,".csv")

write.csv(perm_rt_wuni$aov.tab, file = filename)

##Testing variables - overall

bray_dist = phyloseq::distance(S2_samples, method="bray")

jacc_dist = phyloseq::distance(S2_samples, method="jaccard", binary=TRUE)

unwu_dist = phyloseq::distance(S2_samples, method="unifrac")

wu_dist = phyloseq::distance(S2_samples, method="wunifrac")

#Location of sample

perm_site_b <- adonis(bray_dist ~ sample_data(S2_samples)$'Location.of.sample')

perm_site_b

timestamp_formatted_full <- format(Sys.time(), format="%d-%b-%Y_%H%M")

filename <- paste0(outputs_dir_slash,"PERMANOVA_s2_site_bray_",timestamp_formatted_full,".csv")

write.csv(perm_site_b$aov.tab, file = filename)

perm_site_j <- adonis(jacc_dist ~ sample_data(S2_samples)$'Location.of.sample')

perm_site_j

timestamp_formatted_full <- format(Sys.time(), format="%d-%b-%Y_%H%M")

filename <- paste0(outputs_dir_slash,"PERMANOVA_s2_site_jaccard_",timestamp_formatted_full,".csv")

write.csv(perm_site_j$aov.tab, file = filename)

perm_site_uni <- adonis(unwu_dist ~ sample_data(S2_samples)$'Location.of.sample')

perm_site_uni

timestamp_formatted_full <- format(Sys.time(), format="%d-%b-%Y_%H%M")

filename <- paste0(outputs_dir_slash,"PERMANOVA_s2_site_unweightedUniFrac_",timestamp_formatted_full,".csv")

write.csv(perm_site_uni$aov.tab, file = filename)

perm_site_wuni <- adonis(wu_dist ~ sample_data(S2_samples)$'Location.of.sample')

perm_site_wuni

timestamp_formatted_full <- format(Sys.time(), format="%d-%b-%Y_%H%M")

filename <- paste0(outputs_dir_slash,"PERMANOVA_s2_site_weightedUniFrac_",timestamp_formatted_full,".csv")

write.csv(perm_site_wuni$aov.tab, file = filename)

#Extraction batch

perm_batch_b <- adonis(bray_dist ~ sample_data(ps2_samples)$'Extraction.batch')

perm_batch_b

timestamp_formatted_full <- format(Sys.time(), format="%d-%b-%Y_%H%M")

filename <- paste0(outputs_dir_slash,"PERMANOVA_s2_extraction_batch_bray_",timestamp_formatted_full,".csv")

write.csv(perm_batch_b$aov.tab, file = filename)

perm_batch_j <- adonis(jacc_dist ~ sample_data(ps2_samples)$'Extraction.batch')

perm_batch_j

timestamp_formatted_full <- format(Sys.time(), format="%d-%b-%Y_%H%M")

filename <- paste0(outputs_dir_slash,"PERMANOVA_s2_extraction_batch_jaccard_",timestamp_formatted_full,".csv")

write.csv(perm_batch_j$aov.tab, file = filename)

perm_batch_uni <- adonis(unwu_dist ~ sample_data(ps2_samples)$'Extraction.batch')

perm_batch_uni

timestamp_formatted_full <- format(Sys.time(), format="%d-%b-%Y_%H%M")

filename <- paste0(outputs_dir_slash,"PERMANOVA_s2_extraction_batch_unweightedUniFrac_",timestamp_formatted_full,".csv")

write.csv(perm_batch_uni$aov.tab, file = filename)

perm_batch_wuni <- adonis(wu_dist ~ sample_data(ps2_samples)$'Extraction.batch')

perm_batch_wuni

timestamp_formatted_full <- format(Sys.time(), format="%d-%b-%Y_%H%M")

filename <- paste0(outputs_dir_slash,"PERMANOVA_s2_extraction_batch_weightedUniFrac_",timestamp_formatted_full,".csv")

write.csv(perm_batch_wuni$aov.tab, file = filename)

p15 <- plot_ordination(ps.samples.prop, ord.nmds.bray.sam, title="Bray NMDS - Extraction batch", color="Extraction.batch", shape="Extraction.batch") +

geom_point(size=3)

p15 + stat_ellipse(type="norm", alpha=0.4)

p16 <- plot_ordination(ps.samples.prop, ord.nmds.jac.sam, title="Jaccard NMDS - Extraction batch", color="Extraction.batch", shape="Extraction.batch") +

geom_point(size=3)

p16 + stat_ellipse(type="norm", alpha=0.4)

p17 <- plot_ordination(ps.samples.prop, ps.pcoa.sam, title="Unweighted UniFrac - Extraction batch", color="Extraction.batch", shape="Extraction.batch") +

geom_point(size=3)

p17 + stat_ellipse(type="norm", alpha=0.4)

p18 <- plot_ordination(ps.samples.prop, ps.wpcoa.sam, title="Weighted UniFrac - Extraction batch", color="Extraction.batch", shape="Extraction.batch") +

geom_point(size=3)

p18 + stat_ellipse(type="norm", alpha=0.4)

data_rt <-data.frame(sample_data(S2_rt))

data_rt$Timing <- as.factor(data_rt$Timing)

dim(data_rt)

OTU_rt = as(otu_table(S2_rt), "matrix")

dim(OTU_rt)

adonis(OTU_rt ~ Timing, data=data_rt)

data_4d <-data.frame(sample_data(S2_4d))

data_4d$Timing <- as.factor(data_4d$Timing)

dim(data_4d)

OTU_4d = as(otu_table(S2_4d), "matrix")

dim(OTU_4d)

adonis(OTU_4d ~ Timing, data=data_4d)

perm_test <- adonis2(bray_dist ~ Condition * Timing, data = samp_data)

perm_test

#dispersion measure is bray_dist

S2_mod <-betadisper(bray_dist,sample_data(S2_samples)$SampleLabel)

anova(S2_mod)

TukeyHSD(S2_mod, conf.level = 0.95)

plot(S2_mod)

S2_mod

boxplot(S2_mod, xlab="Sample")

# get betadisper dataframes ####

# adapted from online source - functions to grab the necessary data from the betadisper object

# functions ####

# getting distances from betadisper() object

betadisper_distances <- function(model){

temp <- data.frame(group = model$group)

temp2 <- data.frame(distances = unlist(model$distances))

temp2$sample <- row.names(temp2)

temp <- cbind(temp, temp2)

temp <- dplyr::select(temp, group, sample, dplyr::everything())

row.names(temp) <- NULL

return(temp)

}

# getting eigenvalues out of betadisper() object

betadisper_eigenvalue <- function(model){

temp <- data.frame(eig = unlist(model$eig))

temp$PCoA <- row.names(temp)

row.names(temp) <- NULL

return(temp)

}

# getting the eigenvectors out of a betadisper() object

betadisper_eigenvector <- function(model){

temp <- data.frame(group = model$group)

temp2 <- data.frame(unlist(model$vectors))

temp2$sample <- row.names(temp2)

temp <- cbind(temp, temp2)

temp <- dplyr::select(temp, group, sample, dplyr::everything())

row.names(temp) <- NULL

return(temp)

}

# get centroids

betadisper_centroids <- function(model){

temp <- data.frame(unlist(model$centroids))

temp$group <- row.names(temp)

temp <- dplyr::select(temp, group, dplyr::everything())

row.names(temp) <- NULL

return(temp)

}

# betadisper data

get_betadisper_data <- function(model){

temp <- list(distances = betadisper_distances(model),

eigenvalue = betadisper_eigenvalue(model),

eigenvector = betadisper_eigenvector(model),

centroids = betadisper_centroids(model))

return(temp)

}

# get betadisper data ####

betadisper_dat <- get_betadisper_data(S2_mod)

# do some transformations on the data

betadisper_dat$eigenvalue <- mutate(betadisper_dat$eigenvalue, percent = eig/sum(eig))

betadispersion <- ggplot(betadisper_dat$distances, aes(group, distances, fill = group, col = group)) +

geom_boxplot(fill=NA) +

stat_summary(geom = 'crossbar', width = 0, fun.data = function(x){ return(c(y=median(x), ymin=median(x), ymax=median(x)))}) +

geom_point(aes(group, distances, col = group), shape = 21, position = position_jitterdodge(dodge.width = 0.55, jitter.width = 0.2)) +

theme_bw() +

labs(y='Distance to centroid', x

="\nSamples") +

theme(legend.position = 'none',

axis.text.x = element_text(size=12,angle=270,hjust=0, vjust=0.5),

axis.text.y = element_text(size=12),

axis.title.x = element_text(size=12,color='black'),

axis.title.y = element_text(size=12,color='black'))

timestamp_formatted_full <- format(Sys.time(), format="%d-%b-%Y_%H%M")

filename <- paste0("p_S2_betadispersion_",timestamp_formatted_full,".png")

ggsave(filename, plot=betadispersion, device = png,

path = outputs_dir,

width=16, height=11, units="cm", dpi = 600, limitsize = TRUE)

bray_dist_QC = phyloseq::distance(S2_samples_QC, method="bray")

S2_mod_QC <-betadisper(bray_dist_QC,sample_data(S2_samples_QC)$SampleLabel)

anova(S2_mod_QC)

TukeyHSD(S2_mod_QC)

plot(S2_mod_QC)

S2_mod_QC

boxplot(S2_mod_QC, xlab="Sample")

```

```{stage-two-analysis-bar-plots}

sample_data(S2_4d)$SampleLabel <- as.factor(sample_data(S2_4d)$SampleLabel)

sample_data(S2_4d)$Timing <- as.factor(sample_data(S2_4d)$Timing)

##### 4 degrees - top 100 ASVs #####

top100.4d <- names(sort(taxa_sums(S2_4d), decreasing=TRUE))[1:100]

S2.top100.4d <- prune_taxa(top100.4d, S2.4d.prop100)

xlab = expression(Days~at~"4"*degree*C)

S2_4d_top100_bar <- plot_bar(S2.top100.4d, "Timing", fill= "Genus", facet_grid=~SampleLabel) +

theme_bw() +

theme(axis.text.x = element_text(size=6,angle=270,hjust=0, vjust=0.5),

strip.text.x = element_text(angle=270),

legend.text = element_text(size=9, face="italic"),

legend.key.size = unit(0.4, "cm"),

panel.spacing.x=unit(0.2, "lines"),

legend.margin=margin(0,0,0,0),

legend.box.margin=margin(-5,-5,-5,-5)) +

labs(y = "Relative Abundance (%)", x=xlab) +

geom_bar(stat="identity") +

guides(fill = guide_legend(ncol = 1)) +

scale_x_discrete(labels=c("0 days" = "0 days", "01 day" = "1 day", "05 days" = "5 days", "10 days"="10 days"))

timestamp_formatted_full <- format(Sys.time(), format="%d-%b-%Y_%H%M")

filename <- paste0("p_S2_4d_top100_bar_",timestamp_formatted_full,".png")

ggsave(filename, plot=S2_4d_top100_bar, device = png,

path = outputs_dir,

width=17, height=12.1, units="cm", dpi = 600, limitsize = TRUE)

##### 4 degrees - top 50 ASVs #####

top50.4d <- names(sort(taxa_sums(S2_4d), decreasing=TRUE))[1:50]

S2.top50.4d <- prune_taxa(top50.4d, S2.4d.prop100)

xlab = expression(Days~at~"4"*degree*C)

S2_4d_top50_bar <- plot_bar(S2.top50.4d, "Timing", fill= "Genus", facet_grid=~SampleLabel) +

theme_bw() +

theme(axis.text.x = element_text(size=6,angle=270,hjust=0, vjust=0.5),

strip.text.x = element_text(angle=270),

legend.text = element_text(size=10, face="italic"),

legend.key.size = unit(0.4, "cm"),

panel.spacing.x=unit(0.2, "lines"),

legend.margin=margin(0,0,0,0),

legend.box.margin=margin(-5,-5,-5,-5)) +

labs(y = "Relative Abundance (%)", x=xlab) +

geom_bar(stat="identity") +

guides(fill = guide_legend(ncol = 1)) +

scale_x_discrete(labels=c("0 days" = "0 days", "01 day" = "1 day", "05 days" = "5 days", "10 days"="10 days"))

timestamp_formatted_full <- format(Sys.time(), format="%d-%b-%Y_%H%M")

filename <- paste0("p_S2_4d_top50_bar_",timestamp_formatted_full,".png")

ggsave(filename, plot=S2_4d_top50_bar, device = png,

path = outputs_dir,

width=17.5, height=10, units="cm", dpi = 600, limitsize = TRUE)

##### Room temperature - top 100 ASVs #####

top100.rt <- names(sort(taxa_sums(S2_rt), decreasing=TRUE))[1:100]

S2.top100.rt <- prune_taxa(top100.rt, S2.rt.prop100)

S2_rt_top100_bar <- plot_bar(S2.top100.rt, "Timing", fill= "Genus", facet_grid=~SampleLabel) +

theme_bw() +

theme(axis.text.x = element_text(angle=270,hjust=0, vjust=0.5),

legend.text = element_text(size=9, face="italic"),

legend.key.size = unit(0.3, "cm")) +

labs(y = "Relative Abundance (%)", x="Hours at room temperature") +

geom_bar(stat="identity") +

guides(fill = guide_legend(ncol = 1)) +

scale_x_discrete(labels=c("0 hours" = "0 hours", "04 hours" = "4 hours", "24 hours" = "24 hours", "48 hours"="48 hours"))

timestamp_formatted_full <- format(Sys.time(), format="%d-%b-%Y_%H%M")

filename <- paste0("p_S2_rt_top100_bar_",timestamp_formatted_full,".png")

ggsave(filename, plot=S2_rt_top100_bar, device = png,

path = outputs_dir,

width=17, height=12.1, units="cm", dpi = 600, limitsize = TRUE)

##### Room temperature - top 50 ASVs #####

top50.rt <- names(sort(taxa_sums(S2_rt), decreasing=TRUE))[1:50]

S2.top50.rt <- prune_taxa(top50.rt, S2.rt.prop100)

S2_rt_top50_bar <- plot_bar(S2.top50.rt, "Timing", fill= "Genus", facet_grid=~SampleLabel) +

theme_bw() +

theme(axis.text.x = element_text(angle=270,hjust=0, vjust=0.5),

legend.text = element_text(size=10, face="italic"),

legend.key.size = unit(0.4, "cm")) +

labs(y = "Relative Abundance (%)", x="Hours at room temperature") +

geom_bar(stat="identity") +

guides(fill = guide_legend(ncol = 1)) +

scale_x_discrete(labels=c("0 hours" = "0 hours", "04 hours" = "4 hours", "24 hours" = "24 hours", "48 hours"="48 hours"))

timestamp_formatted_full <- format(Sys.time(), format="%d-%b-%Y_%H%M")

filename <- paste0("p_S2_rt_top50_bar_",timestamp_formatted_full,".png")

ggsave(filename, plot=S2_rt_top50_bar, device = png,

path = outputs_dir,

width=17, height=12.1, units="cm", dpi = 600, limitsize = TRUE)

#Only controls

top100.con <- names(sort(taxa_sums(S2_controls), decreasing=TRUE))[1:100]

S2.top100.con <- prune_taxa(top100.con, S2.controls.prop100)

plot_bar(S2.top100.con, fill="Species") + theme(axis.text.x = element_text(vjust = 0.25)) +labs(y = "Relative Abundance (%)", x="")

plot_bar(S2.top100.con, fill="Genus") + theme(axis.text.x = element_text(vjust = 0.25)) +labs(y = "Relative Abundance (%)", x="")

plot_bar(S2.controls.prop100, fill="Species") + theme(axis.text.x = element_text(vjust = 0.25)) +labs(y = "Relative Abundance (%)", x="")

plot_bar(S2.controls.prop100, fill="Species") + theme(axis.text.x = element_text(vjust = 0.25)) +labs(y = "Relative Abundance (%)", x="")

##Site##

S2.top100 <- names(sort(taxa_sums(S2_samples), decreasing=TRUE))[1:100]

S2.top100.p <- prune_taxa(S2.top100, S2.samples.prop100)

plot_bar(S2.top100.p, fill="Species") + theme(axis.text.x = element_text(vjust = 0.3)) +labs(y = "Relative Abundance (%)")

plot_bar(S2.top100.p, fill="Genus") + theme(axis.text.x = element_text(vjust = 0.3)) +labs(y = "Relative Abundance (%)")

plot_bar(S2.top100.p,"SampleName", fill= "Species", facet_grid=~Location.of.sample) + theme(axis.text.x = element_text(vjust = 0.3)) +labs(y = "Relative Abundance (%)")

plot_bar(S2.top100.p, fill= "Genus", facet_grid=~SampleLabel) + theme(axis.text.x = element_text(vjust = 0.3)) +labs(y = "Relative Abundance (%)")

```

```{stage-two-analysis-rarefaction}

#Rarefy

set.seed(243)

S2_rt_rare <- rarefy_even_depth(S2_rt, sample.size =40000,rngseed = FALSE, replace = TRUE, trimOTUs = TRUE, verbose = TRUE)

set.seed(243)

S2_4d_rare <- rarefy_even_depth(S2_4d, sample.size =40000,rngseed = FALSE, replace = TRUE, trimOTUs = TRUE, verbose = TRUE)

plot_richness(S2_rt_rare, x="Timing", measures = c("Observed","shannon", "simpson"), color = "Timing") + geom_boxplot(fill=NA) + theme(axis.text.x = element_text(vjust = 0.3))

plot_richness(S2_4d_rare, x="Timing", measures = c("Observed","shannon", "simpson"), color = "Timing") + geom_boxplot(fill=NA) + theme(axis.text.x = element_text(vjust = 0.3))

```

```{r deseq2}

library("DESeq2")

packageVersion("DESeq2")

library("XML")

##### 4 degrees #####

#### 0 vs 10 days ####

# prepare data

S2_4d_0_10 <- subset_samples(S2_4d, (Timing == "0 days"| Timing == "10 days"))

S2_4d_0_10 <- prune_taxa(taxa_sums(S2_4d_0_10) > 0, S2_4d_0_10)

S2_4d_0_10

# Make sure timing is a factor

sample_data(S2_4d_0_10)$Timing <- as.factor(sample_data(S2_4d_0_10)$Timing)

#Add an offset of 1 - to avoid the 'every gene contains at least one zero, cannot compute log geometric means' error

otu_table(S2_4d_0_10) <- otu_table(S2_4d_0_10) + 1

#Convert phyloseq object to deseq2

timingdds = phyloseq_to_deseq2(S2_4d_0_10, ~Timing)

timingdds2 <- DESeq(timingdds, test="Wald", fitType="parametric")

timingdds2

res1 = results(timingdds2, cooksCutoff = FALSE)

alpha1 = 0.05

sigtab1 = res1[which(res1$padj < alpha1), ]

sigtab1 = cbind(as(sigtab1, "data.frame"), as(tax_table(S2_4d_0_10)[rownames(sigtab1), ], "matrix"))

head(sigtab1)

Fourdeg_DESeq2_Table <- as.data.frame(sigtab1)

timestamp_formatted_full <- format(Sys.time(), format="%d-%b-%Y_%H%M")

filename <- paste0(outputs_dir_slash,"fourdegrees_deseq_table_10d_",timestamp_formatted_full,".csv")

write.csv(Fourdeg_DESeq2_Table, file = filename)

# Species order

x = tapply(sigtab1$log2FoldChange, sigtab1$Species, function(x) max(x))

x = sort(x, TRUE)

sigtab1$Species = factor(as.character(sigtab1$Species), levels=names(x))

names_update <- c(levels(sigtab1$Species))

names_update

species_label <- c("Peptoniphilus \ngrossensis" ,"Peptoniphilus \nsp.","Veillonella \natypica",

"Gardnerella \nvaginalis","Prevotella \ndisiens", "Anaerosphaera \nsp.",

"Peptostreptococcus \nanaerobius","Bifidobacterium \nbreve","Lactobacillus \njensenii")

names(species_label) <- names_update

v1 <- ggplot(sigtab1, aes(x=Species, y=log2FoldChange, color=Genus)) +

geom_point(size=6) +

theme_bw() +

theme(axis.text.x = element_text(angle=-90,hjust = 0, vjust=0.5, face="italic"),

legend.text = element_text(face="italic")) +

scale_x_discrete(labels=species_label) +

labs(x="\nSpecies")

timestamp_formatted_full <- format(Sys.time(), format="%d-%b-%Y_%H%M")

filename <- paste0("p_S2_4d_0d_vs_10d_deseq2_",timestamp_formatted_full,".png")

ggsave(filename, plot=v1, device = png,

path = outputs_dir,

width=20, height=12.5, units="cm", dpi = 600, limitsize = TRUE)

ASVs_10d <- rownames(Fourdeg_DESeq2_Table)

Gen_4d_10d <- subset_taxa(S2.4d.prop100, rownames(tax_table(S2.4d.prop100)) %in% ASVs_10d)

Gen_4d_10d_sub <- subset_samples(Gen_4d_10d, (Timing == "0 days"| Timing == "10 days"))

my_legend_labels <- c(expression(paste(italic("Anaerosphaera"), " sp.", sep="")),

expression(paste(italic("Bifidobacterium breve"), sep="")),

expression(paste(italic("Gardnerella vaginalis"), sep="")),

expression(paste(italic("Lactobacillus jensenii"), sep="")),

expression(paste(italic("Peptoniphilus grossensis"), sep="")),

expression(paste(italic("Peptostreptococcus anaerobius"), sep="")),

expression(paste(italic("Peptoniphilus"), " sp.", sep="")),

expression(paste(italic("Prevotella disiens"), sep="")),

expression(paste(italic("Veillonella atypica"), sep="")))

bar_0d_10d <- plot_bar(Gen_4d_10d_sub, "SampleLabel", fill="Species") +

theme_bw() +

theme(axis.text.x = element_text(angle=-90,hjust = 0, vjust=0.5,),

strip.text.y = element_text(angle=0, face="italic"),

legend.text.align = 0) +

labs(y = "Relative Abundance (%)", x="") +

facet_grid(rows=Genus~Timing,scales="free_y") +

geom_bar(stat="identity") +

guides(fill = guide_legend(ncol = 1))+

scale_fill_discrete(labels=my_legend_labels)

timestamp_formatted_full <- format(Sys.time(), format="%d-%b-%Y_%H%M")

filename <- paste0("p_0d_vs_10d_bar_plot_",timestamp_formatted_full,".png")

ggsave(filename, plot=bar_0d_10d, device = png,

path = outputs_dir,

width=20, height=27, units="cm", dpi = 600, limitsize = TRUE)

#### 0 vs 5 days ####

S2_4d_0_5 <- subset_samples(S2_4d, (Timing == "0 days"| Timing == "05 days"))

S2_4d_0_5 <- prune_taxa(taxa_sums(S2_4d_0_5) > 0, S2_4d_0_5)

S2_4d_0_5

# Make sure timing is a factor

sample_data(S2_4d_0_5)$Timing <- as.factor(sample_data(S2_4d_0_5)$Timing)

#Add an offset of 1 - to avoid errors with 0's

otu_table(S2_4d_0_5) <- otu_table(S2_4d_0_5) + 1

timingdds3 = phyloseq_to_deseq2(S2_4d_0_5, ~Timing)

timingdds4 <- DESeq(timingdds3, test="Wald", fitType="parametric")

timingdds4

res2 = results(timingdds4, cooksCutoff = FALSE)

alpha = 0.05

sigtab2 = res2[which(res2$padj < alpha), ]

sigtab2 = cbind(as(sigtab2, "data.frame"), as(tax_table(S2_4d_0_5)[rownames(sigtab2), ], "matrix"))

head(sigtab2)

Fourdeg_DESeq2_Table_5d <- as.data.frame(sigtab2)

timestamp_formatted_full <- format(Sys.time(), format="%d-%b-%Y_%H%M")

filename <- paste0(outputs_dir_slash,"fourdegrees_deseq_table_5d_",timestamp_formatted_full,".csv")

write.csv(Fourdeg_DESeq2_Table_5d, file = filename)

# Species order

x = tapply(sigtab2$log2FoldChange, sigtab2$Species, function(x) max(x))

x = sort(x, TRUE)

sigtab2$Species = factor(as.character(sigtab2$Species), levels=names(x))

names_update <- c(levels(sigtab2$Species))

names_update

species_label <- c("Finegoldia \nmagna","Staphylococcus \nhaemolyticus",

"Gardenerella \nvaginalis","Prevotella \ndisiens","Ruminococcus \nfaecis",

"Anaerococcus \nsp.", "Peptoniphilus \nsp.","Corynebacterium \nurealyticum",

"Dialister \nmicraerophilus","Corynebacterium \ncoyleae")

names(species_label) <- names_update

v2 <- ggplot(sigtab2, aes(x=Species, y=log2FoldChange, color=Genus)) + geom_point(size=6) +

theme(axis.text.x = element_text(hjust = 0.5, vjust=0.5)) +

scale_x_discrete(labels=species_label) +

labs(x="\nSpecies")

timestamp_formatted_full <- format(Sys.time(), format="%d-%b-%Y_%H%M")

filename <- paste0("p_S2_4d_0d_vs_5d_deseq2_",timestamp_formatted_full,".png")

ggsave(filename, plot=v2, device = png,

path = outputs_dir,

width=30, height=20, units="cm", dpi = 600, limitsize = TRUE)

ASVs_5d <- rownames(Fourdeg_DESeq2_Table_5d)

Gen_4d_5d <- subset_taxa(S2.4d.prop100, rownames(tax_table(S2.4d.prop100)) %in% ASVs_5d)

Gen_4d_5d_sub <- subset_samples(Gen_4d_50d, (Timing == "0 days"| Timing == "05 days"))

my_legend_labels <- c(expression(paste(italic("Anaerococcus"), " sp.", sep="")),

expression(paste(italic("Corynebacterium coyleae"), sep="")),

expression(paste(italic("Corynebacterium urealyticum"), sep="")),

expression(paste(italic("Dialister microaerophilus"), sep="")),

expression(paste(italic("Finegoldia magna"), sep="")),

expression(paste(italic("Gardnerella vaginalis"), sep="")),

expression(paste(italic("Peptoniphilus"), " sp.", sep="")),

expression(paste(italic("Prevotella disiens"), sep="")),

expression(paste(italic("Ruminococcus faecis"), sep="")),

expression(paste(italic("Staphylococcus haemolyticus"), sep="")))

bar_0d_5d <- plot_bar(Gen_4d_5d_sub, "SampleLabel", fill="Species") + theme(axis.text.x = element_text(vjust = 0.25), strip.text.y = element_text(angle=0, face="italic"), legend.text.align = 0) +

labs(y = "Relative Abundance (%)", x="") +

facet_grid(rows=Genus~Timing,scales="free_y") +

geom_bar(stat="identity") +

guides(fill = guide_legend(ncol = 1))+

scale_fill_discrete(labels=my_legend_labels)

timestamp_formatted_full <- format(Sys.time(), format="%d-%b-%Y_%H%M")

filename <- paste0("p_0d_vs_5d_bar_plot_",timestamp_formatted_full,".png")

ggsave(filename, plot=bar_0d_5d, device = png,

path = outputs_dir,

width=20, height=27, units="cm", dpi = 600, limitsize = TRUE)

#### 0 vs 1 day ####

S2_4d_0_1 <- subset_samples(S2_4d, (Timing == "0 days"| Timing == "01 day"))

S2_4d_0_1 <- prune_taxa(taxa_sums(S2_4d_0_1) > 0, S2_4d_0_1)

S2_4d_0_1

# Make sure timing is a factor

sample_data(S2_4d_0_1)$Timing <- as.factor(sample_data(S2_4d_0_1)$Timing)

#Add an offset of 1 - to avoid errors with 0's

otu_table(S2_4d_0_1) <- otu_table(S2_4d_0_1) + 1

timingdds5 = phyloseq_to_deseq2(S2_4d_0_1, ~Timing)

timingdds6 <- DESeq(timingdds5, test="Wald", fitType="parametric")

timingdds6

res3 = results(timingdds6, cooksCutoff = FALSE)

alpha = 0.05

sigtab3 = res3[which(res3$padj < alpha), ]

sigtab3 = cbind(as(sigtab3, "data.frame"), as(tax_table(S2_4d_0_1)[rownames(sigtab3), ], "matrix"))

head(sigtab3)

Fourdeg_DESeq2_Table_1d <- as.data.frame(sigtab3)

timestamp_formatted_full <- format(Sys.time(), format="%d-%b-%Y_%H%M")

filename <- paste0(outputs_dir_slash,"fourdegrees_deseq_table_1d_",timestamp_formatted_full,".csv")

write.csv(Fourdeg_DESeq2_Table_1d, file = filename)

# Species order

x = tapply(sigtab3$log2FoldChange, sigtab3$Species, function(x) max(x))

x = sort(x, TRUE)

sigtab3$Species = factor(as.character(sigtab3$Species), levels=names(x))

names_update <- c(levels(sigtab3$Species))

names_update

species_label <- c("Finegoldia \nmagna","Campylobacter \nureolyticus","Peptoniphilus \nindolicus",

"Gardenerella \nvaginalis", "Anaerosphaera \nsp.","Anaerococcus \nsp.",

"Anaerococcus \nlactolyticus", "Staphylococcus \nhaemolyticus",

"Corynebacterium \nurealyticum","Moryella \nsp.", "Corynebacterium \ncoyleae",

"Dialister \nmicraerophilus","Fannyhessea \nvaginae","Bifidobacterium \nbreve",

"Corynebacterium \npyruviciproducens")

names(species_label) <- names_update

v3 <- ggplot(sigtab3, aes(x=Species, y=log2FoldChange, color=Genus)) + geom_point(size=6) +

theme(axis.text.x = element_text(angle=-90,hjust = 0, vjust=0.5)) +

scale_x_discrete(labels=species_label) +

labs(x="\nSpecies")

timestamp_formatted_full <- format(Sys.time(), format="%d-%b-%Y_%H%M")

filename <- paste0("p_S2_4d_0d_vs_1d_deseq2_",timestamp_formatted_full,".png")

ggsave(filename, plot=v3, device = png,

path = outputs_dir,

width=30, height=20, units="cm", dpi = 600, limitsize = TRUE)

ASVs_1d <- rownames(Fourdeg_DESeq2_Table_1d)

Gen_4d_1d <- subset_taxa(S2.4d.prop100, rownames(tax_table(S2.4d.prop100)) %in% ASVs_1d)

Gen_4d_1d_sub <- subset_samples(Gen_4d_1d, (Timing == "0 days"| Timing == "01 day"))

my_legend_labels <- c(expression(paste(italic("Anaerococcus"), " sp.", sep="")),

expression(paste(italic("Anaerococcus lactolyticus"), sep="")),

expression(paste(italic("Anaerosphaera"), " sp.", sep="")),

expression(paste(italic("Bifidobacterium breve"), sep="")),

expression(paste(italic("Campylobacter ureolyticus"), sep="")),

expression(paste(italic("Corynebacterium coyleae"), sep="")),

expression(paste(italic("Corynebacterium pyruviciproducens"), sep="")),

expression(paste(italic("Corynebacterium urealyticum"), sep="")),

expression(paste(italic("Dialister microaerophilus"), sep="")),

expression(paste(italic("Fannyhessea vaginae"), sep="")),

expression(paste(italic("Finegoldia magna"), sep="")),

expression(paste(italic("Gardnerella vaginalis"), sep="")),

expression(paste(italic("Moryella"), " sp.", sep="")),

expression(paste(italic("Peptoniphilus indolicus"), sep="")),

expression(paste(italic("Staphylococcus haemolyticus"), sep="")))

bar_0d_1d <- plot_bar(Gen_4d_1d_sub, "SampleLabel", fill="Species") + theme(axis.text.x = element_text(vjust = 0.25), strip.text.y = element_text(angle=0, face="italic"), legend.text.align = 0) +

labs(y = "Relative Abundance (%)", x="") +

facet_grid(rows=Genus~Timing,scales="free_y") +

geom_bar(stat="identity") +

guides(fill = guide_legend(ncol = 1))+

scale_fill_discrete(labels=my_legend_labels)

timestamp_formatted_full <- format(Sys.time(), format="%d-%b-%Y_%H%M")

filename <- paste0("p_0d_vs_1d_bar_plot_",timestamp_formatted_full,".png")

ggsave(filename, plot=bar_0d_1d, device = png,

path = outputs_dir,

width=20, height=27, units="cm", dpi = 600, limitsize = TRUE)

##### Room temperature #####

#### 0 vs 48 hours ####

S2_rt_0_48 <- subset_samples(S2_rt, (Timing == "0 hours"| Timing == "48 hours"))

S2_rt_0_48 <- prune_taxa(taxa_sums(S2_rt_0_48) > 0, S2_rt_0_48)

S2_rt_0_48

#Make sure timing is a factor

sample_data(S2_rt_0_48)$Timing <- as.factor(sample_data(S2_rt_0_48)$Timing)

#Add an offset of 1 - to avoid errors with 0's

otu_table(S2_rt_0_48) <- otu_table(S2_rt_0_48) + 1

# convert to deseq2 object

timingdds7 = phyloseq_to_deseq2(S2_rt_0_48, ~Timing)

timingdds8 <- DESeq(timingdds7, test="Wald", fitType="parametric")

timingdds8

res4 = results(timingdds8, cooksCutoff = FALSE)

alpha = 0.05

sigtab4 = res4[which(res4$padj < alpha), ]

sigtab4 = cbind(as(sigtab4, "data.frame"), as(tax_table(S2_rt_0_48)[rownames(sigtab4), ], "matrix"))

head(sigtab4)

RT_DESeq2_Table_48 <- as.data.frame(sigtab4)

timestamp_formatted_full <- format(Sys.time(), format="%d-%b-%Y_%H%M")

filename <- paste0(outputs_dir_slash,"roomtemp_deseq_table_48h_",timestamp_formatted_full,".csv")

write.csv(RT_DESeq2_Table_48, file = filename)

# Species order

x = tapply(sigtab4$log2FoldChange, sigtab4$Species, function(x) max(x))

x = sort(x, TRUE)

sigtab4$Species = factor(as.character(sigtab4$Species), levels=names(x))

names_update <- c(levels(sigtab4$Species))

names_update

species_label <- c("Streptococcus agalactiae","Escherichia-Shigella sp.","Shigella flexneri",

"Enterococcus faecalis","Escherichia coli","Staphylococcus haemolyticus",

"Staphylococcus condimenti","Staphylococcus epidermidis","Staphylococcus cohnii",

"Staphylococcus hominis","Staphylococcus carnosus","Staphylococcus simulans",

"Peptoniphilus sp.","Schaalia sp.","Schaalia turicensis","Peptoniphilus lacrimalis",

"Prevotella disiens","Anaerococcus hydrogenalis","Peptoniphilus indolicus",

"Dialister propionicifaciens","Prevotella bivia","Actinotignum schaalii",

"Ligilactobacillus animalis","Streptococcus oralis", "Anaerococcus obesiensis",

"Propionimicrobium lymphophilum","Veillonella parvula", "Prevotella timonensis",

"Peptoniphilus coxii","Finegoldia magna","Actinomyces sp.","Bergeyella sp.",

"Varibaculum sp.","Limosilactobacillus vaginalis","Bifidobacterium longum",

"Anaerococcus lactolyticus")

names(species_label) <- names_update

v4 <- ggplot(sigtab4, aes(x=Species, y=log2FoldChange, color=Genus)) + geom_point(size=4) +

theme_bw() +

theme(axis.text.x = element_text(angle=-90,hjust = 0, vjust=0.5, face="italic"),

legend.text = element_text(size=9, face="italic"),

legend.key.size = unit(0.25, "cm"),) +

scale_x_discrete(labels=species_label) +

labs(x="Species")

timestamp_formatted_full <- format(Sys.time(), format="%d-%b-%Y_%H%M")

filename <- paste0("p_S2_rt_0h_vs_48h_deseq2_",timestamp_formatted_full,".png")

ggsave(filename, plot=v4, device = png,

path = outputs_dir,

width=15.88, height=13.5, units="cm", dpi = 600, limitsize = TRUE)

ASVs_48h <- rownames(RT_DESeq2_Table_48)

Gen_rt_48h <- subset_taxa(S2.rt.prop100, rownames(tax_table(S2.rt.prop100)) %in% ASVs_48h)

Gen_rt_48h_sub <- subset_samples(Gen_rt_48h, (Timing == "0 hours"| Timing == "48 hours"))

bar_0h_48h <- plot_bar(Gen_rt_48h_sub, "SampleLabel", fill="Species") + theme(axis.text.x = element_text(vjust = 0.25), strip.text.y = element_text(angle=0, face="italic"), legend.text.align = 0) +

labs(y = "Relative Abundance (%)", x="") +

facet_grid(rows=Genus~Timing,scales="free_y") +

geom_bar(stat="identity") +

guides(fill = guide_legend(ncol = 1))

timestamp_formatted_full <- format(Sys.time(), format="%d-%b-%Y_%H%M")

filename <- paste0("p_0h_vs_48h_bar_plot_",timestamp_formatted_full,".png")

ggsave(filename, plot=bar_0h_48h, device = png,

path = outputs_dir,

width=20, height=27, units="cm", dpi = 600, limitsize = TRUE)

Gen_rt_48h_high <- subset_taxa(Gen_rt_48h_sub, Genus=="Streptococcus" | Genus=="Escherichia-Shigella"|Genus=="Staphylococcus"| Genus=="Enterococcus")

my_legend_labels <- c(expression(paste(italic("Enterococcus faecalis"), sep="")),

expression(paste(italic("Escherichia coli"), sep="")),

expression(paste(italic("Escherichia-Shigella"), " sp.", sep="")),

expression(paste(italic("Shigella flexneri"), sep="")),

expression(paste(italic("Staphylococcus carnosus"), sep="")),

expression(paste(italic("Staphylococcus cohnii"), sep="")),

expression(paste(italic("Staphylococcus condimenti"), sep="")),

expression(paste(italic("Staphylococcus epidermidis"), sep="")),

expression(paste(italic("Staphylococcus haemolyticus"), sep="")),

expression(paste(italic("Staphylococcus hominis"), sep="")),

expression(paste(italic("Staphylococcus simulans"), sep="")),

expression(paste(italic("Streptococcus agalactiae"), sep="")),

expression(paste(italic("Streptococcus oralis"), sep="")))

bar_0h_48h_high <- plot_bar(Gen_rt_48h_high , "SampleLabel", fill="Species") +

theme_bw() +

theme(axis.text.x = element_text(angle=-90,hjust = 0, vjust=0.5),

strip.text.y = element_text(angle=0, face="italic"),

legend.text.align = 0) +

labs(y = "Relative Abundance (%)", x="") +

facet_grid(rows=Genus~Timing,scales="free_y") +

geom_bar(stat="identity") +

guides(fill = guide_legend(ncol = 1))+

scale_fill_discrete(labels=my_legend_labels)

timestamp_formatted_full <- format(Sys.time(), format="%d-%b-%Y_%H%M")

filename <- paste0("p_0h_vs_48h_high_only_bar_plot_",timestamp_formatted_full,".png")

ggsave(filename, plot=bar_0h_48h_high, device = png,

path = outputs_dir,

width=16.5, height=12.5, units="cm", dpi = 600, limitsize = TRUE)

#### 0 vs 24 hours ####

S2_rt_0_24 <- subset_samples(S2_rt, (Timing == "0 hours"| Timing == "24 hours"))

S2_rt_0_24 <- prune_taxa(taxa_sums(S2_rt_0_24) > 0, S2_rt_0_24)

S2_rt_0_24

#Make sure timing is a factor

sample_data(S2_rt_0_24)$Timing <- as.factor(sample_data(S2_rt_0_24)$Timing)

#Add an offset of 1 - to avoid errors with 0's

otu_table(S2_rt_0_24) <- otu_table(S2_rt_0_24) + 1

timingdds9 = phyloseq_to_deseq2(S2_rt_0_24, ~Timing)

timingdds10 <- DESeq(timingdds9, test="Wald", fitType="parametric")

timingdds10

res5 = results(timingdds10, cooksCutoff = FALSE)

alpha = 0.05

sigtab5 = res5[which(res5$padj < alpha), ]

sigtab5 = cbind(as(sigtab5, "data.frame"), as(tax_table(S2_rt_0_24)[rownames(sigtab5), ], "matrix"))

head(sigtab5)

RT_DESeq2_Table_24 <- as.data.frame(sigtab5)

timestamp_formatted_full <- format(Sys.time(), format="%d-%b-%Y_%H%M")

filename <- paste0(outputs_dir_slash,"roomtemp_deseq_table_24h_",timestamp_formatted_full,".csv")

write.csv(RT_DESeq2_Table_24, file = filename)

# Species order

x = tapply(sigtab5$log2FoldChange, sigtab5$Species, function(x) max(x))

x = sort(x, TRUE)

sigtab5$Species = factor(as.character(sigtab5$Species), levels=names(x))

names_update <- c(levels(sigtab5$Species))

names_update

species_label <- c("Enterococcus faecalis","Streptococcus agalactiae","Bifidobacterium longum",

"Staphylococcus haemolyticus","Schaalia sp.","Prevotella disiens",

"Peptoniphilus lacrimalis","Anaerococcus hydrogenalis","Peptoniphilus indolicus",

"Dialister propionicifaciens","Prevotella bivia","Ligilactobacillus animalis",

"Streptococcus oralis","Actinomyces urogenitalis","Veillonella parvula",

"Prevotella timonensis","Anaerococcus lactolyticus","Peptoniphilus coxii",

"Actinomyces sp.","Anaerococcus obesiensis","Limosilactobacillus vaginalis")

names(species_label) <- names_update

v5 <- ggplot(sigtab5, aes(x=Species, y=log2FoldChange, color=Genus)) + geom_point(size=4) +

theme_bw() +

theme(axis.text.x = element_text(angle=-90,hjust = 0, vjust=0.5, face="italic"),

legend.text = element_text(size=9, face="italic"),

legend.key.size = unit(0.25, "cm"),) +

scale_x_discrete(labels=species_label) +

labs(x="Species")

timestamp_formatted_full <- format(Sys.time(), format="%d-%b-%Y_%H%M")

filename <- paste0("p_S2_rt_0h_vs_24h_deseq2_",timestamp_formatted_full,".png")

ggsave(filename, plot=v5, device = png,

path = outputs_dir,

width=15.88, height=13.5, units="cm", dpi = 600, limitsize = TRUE)

ASVs_24h <- rownames(RT_DESeq2_Table_24)

Gen_rt_24h <- subset_taxa(S2.rt.prop100, rownames(tax_table(S2.rt.prop100)) %in% ASVs_24h)

Gen_rt_24h_sub <- subset_samples(Gen_rt_24h, (Timing == "0 hours"| Timing == "24 hours"))

my_legend_labels <- c(expression(paste(italic("Actinomyces"), " sp.", sep="")),

expression(paste(italic("Actinomyces urogenitalis"), sep="")),

expression(paste(italic("Anaerococcus hydrogenalis"), sep="")),

expression(paste(italic("Anaerococcus lactolyticus"), sep="")),

expression(paste(italic("Anaerococcus obesiensis"), sep="")),

expression(paste(italic("Bifidobacterium longum"), sep="")),

expression(paste(italic("Dialister propionicifaciens"), sep="")),

expression(paste(italic("Enterococcus faecalis"), sep="")),

expression(paste(italic("Ligilactobacillus animalis"), sep="")),

expression(paste(italic("Limosilactobacillus vaginalis"), sep="")),

expression(paste(italic("Peptoniphilus coxii"), sep="")),

expression(paste(italic("Peptoniphilus indolicus"), sep="")),

expression(paste(italic("Peptoniphilus lacrimalis"), sep="")),

expression(paste(italic("Prevotella bivia"), sep="")),

expression(paste(italic("Prevotella disiens"), sep="")),

expression(paste(italic("Prevotella timonensis"), sep="")),

expression(paste(italic("Schaalia"), " sp.", sep="")),

expression(paste(italic("Staphylococcus haemolyticus"), sep="")),

expression(paste(italic("Streptococcus agalactiae"), sep="")),

expression(paste(italic("Streptococcus oralis"), sep="")),

expression(paste(italic("Veillonella parvula"), sep="")))

bar_0h_24h <- plot_bar(Gen_rt_24h_sub, "SampleLabel", fill="Species") + theme(axis.text.x = element_text(vjust = 0.25), strip.text.y = element_text(angle=0, face="italic"), legend.text.align = 0) +

labs(y = "Relative Abundance (%)", x="") +

facet_grid(rows=Genus~Timing,scales="free_y") +

geom_bar(stat="identity") +

guides(fill = guide_legend(ncol = 1))+

scale_fill_discrete(labels=my_legend_labels)

timestamp_formatted_full <- format(Sys.time(), format="%d-%b-%Y_%H%M")

filename <- paste0("p_0h_vs_24h_bar_plot_",timestamp_formatted_full,".png")

ggsave(filename, plot=bar_0h_24h, device = png,

path = outputs_dir,

width=20, height=27, units="cm", dpi = 600, limitsize = TRUE)

#### 0 vs 4 hours ####

S2_rt_0_4 <- subset_samples(S2_rt, (Timing == "0 hours"| Timing == "04 hours"))

S2_rt_0_4 <- prune_taxa(taxa_sums(S2_rt_0_4) > 0, S2_rt_0_4)

S2_rt_0_4

#make sure timing is a factor

sample_data(S2_rt_0_4)$Timing <- as.factor(sample_data(S2_rt_0_4)$Timing)

#Add an offset of 1 - to avoid issues with 0's

otu_table(S2_rt_0_4) <- otu_table(S2_rt_0_4) + 1

timingdds11 = phyloseq_to_deseq2(S2_rt_0_4, ~Timing)

timingdds12 <- DESeq(timingdds11, test="Wald", fitType="parametric")

timingdds12

res6 = results(timingdds12, cooksCutoff = FALSE)

alpha = 0.05

sigtab6 = res6[which(res6$padj < alpha), ]

sigtab6 = cbind(as(sigtab6, "data.frame"), as(tax_table(S2_rt_0_4)[rownames(sigtab6), ], "matrix"))

head(sigtab6)

RT_DESeq2_Table_4 <- as.data.frame(sigtab6)

timestamp_formatted_full <- format(Sys.time(), format="%d-%b-%Y_%H%M")

filename <- paste0(outputs_dir_slash,"roomtemp_deseq_table_4h_",timestamp_formatted_full,".csv")

write.csv(RT_DESeq2_Table_4, file = filename)

### no differences found for 4 hours ###

```

```{r random-forest}

library(devtools)

library(randomForest)

library(plyr) # for the "arrange" function

library(caret) # to get leave-one-out cross-validation accuracies and also contains the nearZeroVar function

# rfUtilities has been removed from cran - can be installed directly from the downloaded tar.gz archive file (v2.1-5)

#install.packages(paste(current_dir,"/rfUtilities_2.1-5.tar.gz", sep=""), repos = NULL, type="source")

library(rfUtilities) # to test model significance

sample_data(S2.samples.prop100)$Location.of.sample <- as.factor(sample_data(S2.samples.prop100)$Location.of.sample)

set.seed(151)

RF_site <- randomForest(sample_data(S2.samples.prop100)$Location.of.sample ~ ., data=otu_table(S2.samples.prop100), localImp=TRUE)

RF_site

importance(RF_site)

RF_votes <- as.data.frame(RF_site$votes)

timestamp_formatted_full <- format(Sys.time(), format="%d-%b-%Y_%H%M")

filename <- paste0(outputs_dir_slash,"randomForest_votes_",timestamp_formatted_full,".csv")

write.csv(RF_votes, file = filename)

names(RF_site)

RF_state_site_sig <- rf.significance( x=RF_site , xdata=otu_table(S2.samples.prop100) , nperm=500 , ntree=501 )

RF_state_site_sig

RF_state_site_sig$pValue

timestamp_formatted_full <- format(Sys.time(), format="%d-%b-%Y_%H%M")

filename <- paste0(outputs_dir_slash,"randomForest_significance_",timestamp_formatted_full,".csv")

write.csv(RF_state_site_sig, file = filename)

RF_site_imp <- as.data.frame(importance(RF_site))

RF_site_imp_sorted <- RF_site_imp[order(-RF_site_imp$MeanDecreaseGini),]

RF_site_imp_sorted$ASVs <- row.names(RF_site_imp_sorted)

timestamp_formatted_full <- format(Sys.time(), format="%d-%b-%Y_%H%M")

filename <- paste0(outputs_dir_slash,"randomForest_importance_",timestamp_formatted_full,".csv")

write.csv(RF_site_imp_sorted, file = filename)

# Look at only the top 20 features/ASVs

RF_site_imp_top20 <- rownames(head(RF_site_imp_sorted,20))

RF_site_imp_top20

# Get taxonomy for the ASVs

RF_imp_top20 <- subset_taxa(S2.samples.prop100, rownames(tax_table(S2.samples.prop100)) %in% RF_site_imp_top20)

RF_imp_top20_tempdf <- data.frame(ASVs=rownames(tax_table(RF_imp_top20)),tax_table(RF_imp_top20))

RF_site_imp_top20 <- merge(RF_site_imp_sorted, RF_imp_top20_tempdf, by="ASVs",all = FALSE)

# Get a better label for the x-axis

RF_site_imp_top20$Label <- 0

for (i in 1:nrow(RF_site_imp_top20)){

species_label <- RF_site_imp_top20[i,'Species']

species_name = grepl("_sp",species_label)

if (species_name==TRUE){

genus <- RF_site_imp_top20[i,'Genus']

label <- paste(genus, "sp.", sep=" ")

}

if (species_name==FALSE){

species <- str_split(species_label, '_', simplify = TRUE)[,2]

genus <- RF_site_imp_top20[i,'Genus']

label <- paste(genus,species, sep=" ")

}

RF_site_imp_top20[i,'Label'] <- label

}

# Sort by descending order of variable importance

RF_site_imp_top20_sorted <- RF_site_imp_top20[order(-RF_site_imp_top20$MeanDecreaseGini),]

# Plot the top 20

my_x_axis_labels <- c(RF_site_imp_top20_sorted$Label)

rf_import_site <- ggplot(RF_site_imp_top20_sorted, aes(x=reorder(ASVs,-MeanDecreaseGini),y=MeanDecreaseGini)) +

geom_col() +

labs(y="Mean Decrease in Gini (Variable Importance)",x="") +

scale_y_continuous(limits = c(0, 2.5),expand = c(0, 0)) +

theme_bw() +

scale_x_discrete(labels=my_x_axis_labels) +

theme(axis.text.x = element_text(angle=-90, face="italic", hjust = 0, vjust=0.5),

panel.grid = element_blank(),

panel.border = element_blank(),

axis.ticks.x = element_blank(),

axis.line.y.left = element_line(color="black", lineend="square"))

timestamp_formatted_full <- format(Sys.time(), format="%d-%b-%Y_%H%M")

filename <- paste0("p_random_forest_importance_",timestamp_formatted_full,".png")

ggsave(filename, plot=rf_import_site, device = png,

path = outputs_dir,

width=16, height=15, units="cm", dpi = 600, limitsize = TRUE)

plot_heatmap(RF_imp_top20, method=NULL, taxa.label ="Species", sample.order ="Location.of.sample")

#Try prediction of stage one

Site_pred_S2 <- predict(RF_site, otu_table(S2_samples))

Site_pred_S2

S1.noprune <- subset_samples(ps2, Stage == "One")

S1_samples.noprune <- subset_samples(S1.noprune, Sample.Type == "Sample")

RF_site$type

Site_pred_S1 <- predict(RF_site, otu_table(S1_samples.noprune))

Site_pred_S1

sample_data(S1_samples)$Location.of.sample <- factor(Site_pred_S1)

# Does this data fit similar trends in stage two samples?

plot_richness(S1_samples, x="Location.of.sample", measures = c("Observed","shannon", "simpson"), color = "Location.of.sample") + geom_boxplot(fill=NA)

```

```{r session-info}

sessionInfo()

```
